# Quality of Life after Diet or Exercise-Induced Weight Loss in Overweight to Obese Postmenopausal Women: The SHAPE-2 Randomised Controlled Trial

**DOI:** 10.1371/journal.pone.0127520

**Published:** 2015-06-01

**Authors:** Willemijn A. M. van Gemert, Job van der Palen, Evelyn M. Monninkhof, Anouk Rozeboom, Roelof Peters, Harriet Wittink, Albertine J. Schuit, Petra H. Peeters

**Affiliations:** 1 University Medical Center Utrecht, Julius Center for Health Sciences and Primary Care, department of epidemiology, P.O. Box 85500, 3508 GA, Utrecht, the Netherlands; 2 Medisch Spectrum Twente Hospital, Department of Epidemiology, P.O. Box 50000, 7500 KA, Enschede, the Netherlands; 3 University of Twente, Department of Research Methodology, Measurement, and Data Analysis, P.O. Box 217, 7500 AE, Enschede, the Netherlands; 4 Utrecht University of Applied Sciences, Research group Lifestyle and Health, Faculty of Health Care, P.O. Box 85182, 3508 AD, Utrecht, the Netherlands; 5 National Institute for Public Health and the Environment, Division of Public Health and Health Care, P.O. Box 1, 3720 BA, Bilthoven, the Netherlands; 6 VU University, Department of Health Sciences and EMGO Institute for Health and Care Research, Van der Boechorststraat 7, 1081 BT, Amsterdam, the Netherlands; University of Alabama at Birmingham, UNITED STATES

## Abstract

**Introduction:**

This study investigates the effect of a modest weight loss either by a calorie restricted diet or mainly by increased physical exercise on health related quality of life (HRQoL) in overweight-to-obese and inactive postmenopausal women. We hypothesize that HRQoL improves with weight loss, and that exercise-induced weight loss is more effective for this than diet-induced weight loss.

**Methods:**

The SHAPE-2 trial was primarily designed to evaluate any additional effect of weight loss by exercise compared with a comparable amount of weight loss by diet on biomarkers relevant for breast cancer risk. In the present analysis we focus on HRQoL. We randomly assigned 243 eligible women to a diet (n = 97), exercise (n = 98), or control group (n = 48). Both interventions aimed for 5–6 kg weight loss. HRQoL was measured at baseline and after 16 weeks by the SF-36 questionnaire.

**Results:**

Data of 214 women were available for analysis. Weight loss was 4.9 kg (6.1%) and 5.5 kg (6.9%) with diet and exercise, respectively. Scores of the SF-36 domain *‘health change’* increased significantly by 8.8 points (95% CI 1.6;16.1) with diet, and by 20.5 points (95% CI 13.2;27.7) with exercise when compared with control. Direct comparison of diet and exercise showed a statistically significantly stronger improvement with exercise. Both intervention groups showed a tendency towards improvements in most other domains, which were more pronounced in the exercise group, but not statistically different from control or each other.

**Conclusion:**

In a randomized trial in overweight-to-obese and inactive postmenopausal women a comparable 6%-7% weight loss was achieved by diet-only or mainly by exercise and showed improvements in physical and mental HRQoL domains, but results were not statistically significant in either the diet or exercise group. However, a modest weight loss does lead to a positive change in self-perceived health status. This effect was significantly larger with exercise-induced weight loss than with comparable diet-induced weight loss.

**Trial Registration:**

ClinicalTrials.gov NCT01511276

## Introduction

Obesity is a growing global public health problem [1]. According to the World Health Organisation, the worldwide prevalence of obesity has doubled since 1980 [2]. Obese individuals are at an increased risk of chronic diseases such as diabetes, cardiovascular diseases and cancer [2]. Furthermore, obesity has been associated with a lower health related quality of life (HRQoL) [3]. Negative effects on quality of life are more pronounced in women than men [4]. And since postmenopausal breast cancer is related to obesity[5], and breast cancer worldwide is the most frequent cancer type [6], this age group is a relevant population to study.

A review evaluating nine studies in postmenopausal women found that obese women report a lower HRQoL compared with their lean counterparts [7]. Lifestyle interventions inducing weight loss may therefore provide a self-evident option to improve HRQoL in an overweight to obese population. However, even though some trials observed improvements in HRQoL after reduction in body weight, others did not [8].

Little is known about whether positive effects on quality of life are due to the weight loss or due to the related change in lifestyle [9]. Exercise seems to have a positive influence on HRQoL, also independent of related weight loss [10–13].

The Sex Hormones and Physical Exercise (SHAPE)-2 study was designed to investigate the effect of weight loss, with or without exercise, on health outcomes related to breast cancer risk in postmenopausal women. In this paper, we address the effects on HRQoL. We hypothesise that modest weight loss results in improvements in HRQoL and that effects are larger when weight loss is mainly achieved by exercise compared with an equivalent weight loss induced by diet only.

## Methods

The SHAPE-2 study is a three-armed randomised controlled trial that was conducted in eight municipalities surrounding two research centres in the Netherlands from February 2012 to May 2013. Detailed methods have been described elsewhere [14].

In short, women were recruited from the general population by mass mailings and media attention. Inclusion criteria were ages 50–69 years; postmenopausal (>12 months after last menses); body mass index (BMI) 25–35 kg/m^2^; an inactive lifestyle (<2 hours per week of at least moderate intensity activities, ≥4 metabolic equivalent (MET)). Main exclusion criteria were use of sex hormones; diabetes; smoking; ever diagnosed with breast cancer or other cancer types in the past five years.

All women started with a four to six-week run-in period, wherein a standardised diet was prescribed aiming to stabilise body weight and achieve similarity in diet composition among the study participants [14]. The run-in diet was based on the Dutch Guidelines for a Healthy diet (50%-60% carbohydrates, 15%-20% proteins and 20%-35% fat) [15]. The total amount of calories prescribed meets the individuals energy requirements to maintain stable weight, based on a dietary history, energy calculation by the Harris & Benedict formula [16] and physical activity level. In the intervention period, 243 women were randomised by computer, stratified for municipality to a diet group (n = 97); an exercise group (n = 98) or a waiting list control group (n = 48). The computer software randomized in block sizes of five with a ratio of 2:2:1 for both intervention groups and control, respectively. The aim for women in both intervention groups was to lose 5–6 kg of weight. After the weight loss target was reached, or after a maximum of 14 weeks, women entered a maintenance period wherein stable weight was aimed for.

### Ethics Statement

The study was approved by the ethical committee of the University Medical Center Utrecht. Written informed consent was obtained during the screening visit from all participants.

### Interventions

The diet group intervention consisted of an energy restricted diet (-500 kcal/day). Dietary composition was comparable to the run-in diet. The diet intervention was provided by a dietitian and consisted of two individual 30 minutes sessions, five one-hour interactive group sessions and eight follow-up telephone calls. The sessions consisted of nutrition education, behaviour change techniques and self-management training [17,18]. Women were requested to maintain their habitual physical activity level.

The exercise group followed a 16-week combined aerobic and strength exercise programme. The programme consisted of four hours per week moderate-to-vigorous intensity exercise, resulting in an estimated average energy expenditure of 350 kcal/day. Per week, two one-hour fitness group sessions at a physiotherapist centre were scheduled (60%-95% of the heart rate reserve (HRR)) and two additional hours of Nordic walking (60%-65% HRR). Participants in the exercise group also received an energy intake restriction of -250 kcal/day during the weight loss period and diet composition was also comparable to the run-in diet. During the maintenance period, women continued exercising and their diet was adjusted to meet their energy needs in order to keep their weight stable.

Women in the control group were requested to retain their body weight by continuing the run-in diet and to maintain their habitual exercise level. Controls were offered an alternative weight loss programme after completion of the study.

### Outcomes and measurements

Study participants visited the research centre for measurements at baseline (i.e. randomisation) and after 16 weeks of intervention. Information on sociodemographic variables and general health were assessed by questionnaires. Anthropometric measures were taken according to standard procedures. A total body dual-energy X-ray absorptiometry (DEXA) scan (Lunar, Prodigy) was performed to measure body composition (fat and lean mass). Current level of physical activity was measured by the SQUASH questionnaire. The SQUASH is a short questionnaire assessing habitual physical activity during a normal week over the past few months. The questionnaire measures physical activity in the following domains: commuting activities, household activities, leisure-time and sports activities, and activities at work and school. The SQUASH is fairly reliable and reasonably valid in ordering adults according to their level of physical activity and has a Spearman correlation for overall reproducibility of 0.58 (95%-CI 0.36–0.74) in adults [19].

Cardio respiratory fitness, expressed as VO_2peak_, was measured by performing a maximal cycle exercise test. VO_2peak_ is described as the highest 15-second average of VO_2_ uptake at the end of the test period.

HRQoL was assessed by the 36-item Short-Form Health Survey (SF-36) [20]. The SF-36 is a generic questionnaire to measure health status and consists of eight dimensions: physical functioning (10 items); role limitations by physical problems (4 items); bodily pain (2 items); general health perceptions (5 items); vitality (4 items); social role functioning (2 items); role limitations by emotional problems (3 items) and mental health (5 items). For each dimension, a weighted total score is calculated, ranging from 0 to 100. A higher score on the scale indicates a better health status [20]. Based on the 8 domains, two component summary scores are calculated, i.e. the Physical Component Score (PCS, first 5 domains) and a Mental Component Score (MCS, last 5 domains) [21]. The domain ‘vitality’ and ‘general health’ are both included in the PCS and MCS. The scores are sex-standardised and represent health scores of, in our case, the Dutch population with a mean of 50, and a standard deviation of 10. For example, a PCS score of 60 indicates that physical health is improved with one standard deviation in our study population compared to the general female population.

An additional question in the SF-36 is on ‘*health change’* (1 item) and is not included in the summary scores. It asks participants to report on their self-perceived health by comparing it to one year ago [20]. The range is from 0 to 100, and a score of 50 means no change in perceived health. A score below 50 denotes deterioration, while scores over 50 represent improvement.

Domains of the SF-36 have a high internal consistency: Cronbach’s α range from 0.71 to 0.93 in a Dutch population [22] and were over 0.8 in an elderly population [23]. The construct validity ranges from 0.31 to 0.71 [22].

### Statistical analyses

Sample size calculations were based on the primary outcome of the SHAPE-2 trial, i.e. serum estradiol [14]. Descriptive statistics are presented as mean and standard deviation or median and interquartile range. The main analyses are performed according to the intention-to-treat principle. Complete cases, i.e. women who filled in a questionnaire both at baseline and end-of-study, are included and presented. A paired T-test was used to compute within-group differences. Linear regression analysis was used to investigate between-group differences in SF-36 scores per domain and were adjusted for the baseline SF-36 score. Regression coefficients (β) with 95% confidence intervals (95%CI) are presented. Estimates of effect sizes by Cohen’s *d* were calculated for the regression coefficients. Cohen’s *d* represents the standardised mean difference between two group means. An effect size of 0.5 therefore indicates that the group mean was 0.5 standard deviations higher than the reference group, i.e. the control group, or the diet group in the comparison exercise versus diet [24]. Values below 0.2 are considered small, around 0.5 are considered medium, and large when over 0.8.

Additional analyses were done to study effects of weight loss and change in fitness (as independent variables), regardless of the group assignment. Results are presented as standardised regression coefficients (St-b), representing the effect of change in one standard deviation (SD) of change in weight of fitness, on the change in SDs of HRQOL. Bi-variable and multivariable regression analyses were used. Multivariable models were adjusted for intervention group, age, education, baseline weight and baseline SF-36 score (model 2). Additionally, the model was adjusted for change in physical fitness (VO_2peak_) or change in weight (depending on the determinant of interest, i.e., change in weight or physical fitness, respectively) (model 3).

All analyses were performed using SPSS software (version 21.0). An alpha level of 0.05 for two-sided tests was chosen as significant.

## Results

**Fig 1:**
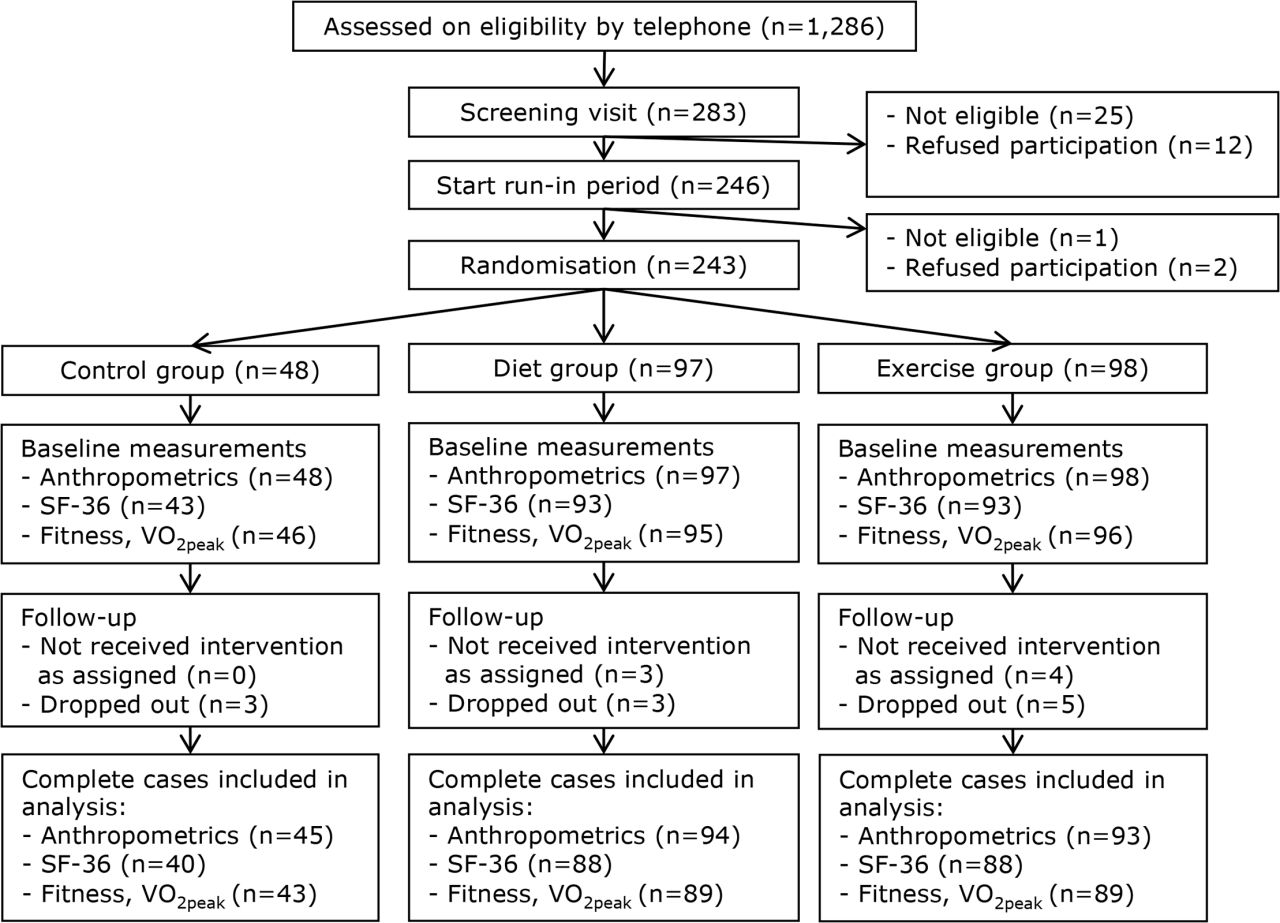
Flow-chart of the inclusion, random assignment, and follow-up of the SHAPE-2 study participants.

In total, 243 women were randomised (Fig 1) of which complete case HRQoL data of 214 (88.1%) women was available and used for the current analysis. Participants were on average 60 years old, had a body weight of 80 kg and had a BMI of 29.2 kg/m^2^ (Table 1). There were no differences in baseline characteristics between the study groups.

**Table 1:** Baseline characteristics of the SHAPE-2 study participants.

	Control group	Diet group	Exercise group
	(n = 48)	(n = 97)	(n = 98)
	*Mean (standard deviation)*		
Age (years)	60.0 (4.9)	60.5 (4.6)	59.5 (4.9)
Time since last menses (years)	11.4 (7.8)	10.7 (6.1)	10.9 (7.7)
Education*, number (%)			
Low	15 (31.3%)	27 (27.8%)	33 (33.6%)
Middle	15 (31.3%)	27 (27.8%)	20 (20.4%)
High	18 (37.5%)	42 (43.3%)	44 (44.9%)
Married or living with partner, number (%)	37 (77.1%)	77 (81.1%)	73 (75.3%)
Weight (kg)	80.9 (10.0)	80.0 (8.6)	80.4 (9.0)
BMI (kg/m^2^)	29.5 (2.6)	29.3 (2.5)	29.0 (2.9)
Body fat percentage (%)	43.6 (5.0)	44.1 (3.8)	43.8 (4.0)
Total body fat (kg)	34.2 (7.4)	33.9 (5.7)	33.9 (6.2)
Lean mass (kg)	43.4 (3.9)	42.7 (4.0)	43.1 (4.1)
VO_2peak,_ relative (ml/kg/min)	22.1 (4.7)	21.9 (4.0)	21.8 (3.7)
VO_2peak_ (mL/min)	1751 (363)	1742 (310)	1749 (293)
Physical activity, by activity monitor† (min/day)	*Median (interquartile range)*		
Sedentary	652 (600–691)	637 (606–685)	630 (593–678)
Light	179 (164–226)	194 (175–214)	197 (157–229)
Moderate	35 (25–39)	35 (22–46)	33 (27–46)
Vigorous	0.3 (0.2–0.6)	0.4 (0.2–0.5)	0.3 (0.1–0.5)
SQUASH moderate and vigorous activity‡ (min/wk)	270 (120–495)	184 (115–420)	248 (90–465)
*SF-36 domains*	*Mean (standard deviation)*		
1-Physical functioning	89.0 (10.3)	85.1 (15.2)	86.9 (10.5)
2-Role-physical	89.5 (24.5)	84.7 (31.7)	87.8 (27.4)
3-Bodily pain	84.1 (20.3)	83.3 (19.3)	82.9 (18.4)
4-General health	72.9 (14.4)	71.3 (14.4)	72.0 (14.4)
5-Vitality	69.3 (12.5)	68.9 (15.8)	69.8 (14.9)
6-Social functioning	88.4 (16.5)	87.5 (17.1)	87.4 (16.5)
7-Role-emotional	95.4 (18.7)	87.8 (30.2)	90.6 (25.8)
8-Mental health	78.2 (15.6)	79.6 (12.2)	77.2 (12.9)
PCS	53.5 (5.2)	52.3 (7.3)	53.1 (6.1)
MCS	52.8 (6.8)	52.7 (8.1)	52.2 (8.0)
Health change	52.3 (17.9)	51.1 (16.9)	50.3 (13.0)

PCS: Physical Component Score (1–5). MCS: Mental Component Score (4–8). NOTE 1. Data available for: VO_2peak_ n = 237 (97.5%); alcohol intake, n = 226 (93.0%); SQUASH physical activity questionnaire, n = 236 (97.1%); ActiGraph accelerometer, n = 161 (out of 215 (74.9%)). For SF-36: domains social functioning, *health change*, and physical and mental component summary score, n = 229 (94.2%, control group, n = 43; diet group, n = 93; exercise group, n = 93); for physical functioning, role-physical and role-emotional, n = 228 (93.8%); and bodily pain, n = 227 (93.4%). NOTE 2. The eight SF-36 domain scores range from 0 to 100, a higher score on the scale indicates a better health status. The score of the domain ‘*health change’* ranges from 0 to 100, a score of 50 means no change in perceived health, a score <50 denotes deterioration and >50 improvement. The summary scores PCS and MCS are sex-standardised and represent health scores of the Dutch population with a mean of 50, and a standard deviation (SD) of 10. For example, a PCS score of 60 indicates that physical health is improved with one SD in our study population compared to the general female population.

* Education, low: primary school and technical/professional school. Middle: college degree. High: university degree

† GT3X+ ActiGraph activity monitor. Minutes/day of activity spent in each activity category. Activity categories are based on Freedson 1998 cutoff points.

‡Based on the SQUASH physical activity questionnaire, activities performed ≥4 METs.

The results of the SHAPE-2 trial on body composition and fitness will be published separately. In short, the diet and the exercise group both lost the aimed body weight (-4.9, 95%CI -5.4 to -4.4, and -5.5 kg, 95%CI -5.9 to -5.1, respectively), while the control group remained weight stable (+0.06 kg, 95%CI -0.34 to -0.46). Body fat percentage changed by -4.1% (95%CI -4.6 to -3.7) in the exercise group, by -2.5% (95%CI -3.0 to -2.1) in the diet group and by +0.2% (95%CI -0.2 to 0.6) in controls. Fitness (VO_2peak_), increased significantly in the exercise group (+119 mL/min, 95%CI 80.5 to 157.0), and showed non-significant decreases with diet (-44.9 mL/min, 95%CI -82.5 to -7.4) and control (-78.6 mL/min, 95%CI -133.3 to -23.8). Moderate to vigorous physical activity according to the SQUASH questionnaire also increased in the exercise group by 222 min/week (95% CI 43;401) compared to control and by 304 (95% CI 158;451) compared to diet. The median number of group sessions attended by women in the diet group was four (out of five offered). In the exercise group, the median attendance of all exercise sessions was 84%. No serious adverse events occurred.

### Intervention effects on HRQoL domains

Baseline domain scores of the SF-36 were comparable in the three study groups (Table 1). At baseline scores for physical functioning, role physical and role emotional were slightly higher in the control group compared with both intervention groups.

Except for *health change*, no significant differences in change in HRQoL were observed when comparing the intervention groups to control (Table 2). Only self-perceived health (domain *health change)* increased significantly in both intervention groups versus control (regression coefficient (β) = 8.8, 95%CI 1.5 to 16.1, for diet and β = 20.5, 95%CI 13.2 to 27.7, for exercise). The improvement was larger in the exercise group when directly compared to diet (β = 11.7, 95%CI 5.9 to 17.4). Also for all other domains, the exercise group showed larger improvements when compared with the diet group, except for general health. However, none of these differences were statistically significant.

**Table 2:** Baseline and 16-week differences in HRQoL and intervention effects between study groups.

	Baseline mean (SD)	16 weeks mean (SD)	Change 16 weeks (95% CI)	Treatment effect (95% CI): Intervention vs Control	P-value	Cohen’s *d* effect size	Treatment effect (95% CI): Exercise vs Diet	P-value	Cohen’s *d* effect size
1-Physical functioning (n = 211)									
Control	90.0 (9.5)	90.0 (11.5)	0.0 (-2.8;2.8)						
Diet	85.6 (15.1)	87.9 (13.8)	2.3 (-0.1;4.5)	0.77 (-2.74;4.28)	0.67	0.08			
Exercise	87.0 (10.5)	91.0 (11.1)	4.0 (1.8;6.1)	2.97 (-0.51;6.46)	0.09	0.33	2.20 (-0.55;4.95)	0.12	0.24
2-Role-physical (n = 211)									
Control	91.0 (21.1)	89.1 (27.4)	-1.9 (-12;8.2)						
Diet	85.9 (30.2)	87.1 (29.0)	1.2 (-6.9;9.2)	-0.91 (-11.0;9.17)	0.86	0.03			
Exercise	88.2 (26.4)	91.4 (24.7)	3.2 (-3.5;9.8)	2.89 (-7.14;12.93)	0.57	0.11	3.81 (-4.13;11.8)	0.35	0.15
3-Bodily pain (n = 208)									
Control	86.7 (18.7)	89.7 (14.7)	3.0 (-3.8;9.8)						
Diet	85.0 (17.8)	83.5 (21.7)	-1.5 (-6.1;3.2)	-5.41 (-11.8;1.00)	0.10	0.33			
Exercise	83.4 (17.9)	86.4 (16.6)	3.0 (-0.6;6.6)	-1.88 (-8.27;4.51)	0.56	0.11	3.53 (-1.55;8.62)	0.17	0.21
4-General health (n = 209)									
Control	73.8 (13.8)	73.4 (14.3)	-0.4 (-4.5;3.7)						
Diet	71.8 (14.4)	74.8 (14.7)	3.0 (0.6;5.4)	2.77 (-1.48;7.01)	0.20	0.26			
Exercise	72.0 (14.7)	74.7 (14.8)	2.7 (0.1;5.3)	2.52 (-1.69;6.74)	0.24	0.23	-0.24 (-3.52;3.04)	0.88	0.02
5-Vitality (n = 210)									
Control	71.2 (11.0)	70.7 (14.7)	-0.5 (-3.9;2.8)						
Diet	69.4 (15.6)	70.5 (17.1)	1.0 (-2.2;4.2)	0.86 (-4.22;5.95)	0.74	0.07			
Exercise	69.8 (15.1)	72.3 (15.0)	2.5 (-0.7;5.7)	2.46 (-2.60;7.51)	0.34	0.19	1.59 (-2.33;5.52)	0.42	0.12
6-Social functioning (n = 214)									
Control	90.1 (16.0)	90.4 (16.1)	0.3 (-5.6;6.2)						
Diet	88.1 (16.7)	86.2 (20.5)	-1.9 (-6.1;2.4)	-3.37 (-9.49;2.75)	0.28	0.21			
Exercise	87.2 (16.8)	89.1 (14.4)	1.9 (-1.9;5.6)	-0.16 (-6.27;5.96)	0.96	0.01	3.21 (-1.59;8.00)	0.19	0.20
7-Role-emotional (n = 210)									
Control	97.4 (11.8)	95.7 (13.6)	-1.7 (-4.1;0.7)						
Diet	87.7 (30.1)	89.3 (29.8)	1.6 (-6.7;9.9)	-3.65 (-12.4;5.05)	0.41	0.16			
Exercise	90.0 (26.5)	93.1 (20.4)	3.1 (-2.2;8.3)	-0.50 (-9.13;8.12)	0.91	0.02	3.15 (-3.67;9.96)	0.36	0.14
8-Mental health (n = 210)									
Control	80.3 (14.0)	79.9 (12.4)	-0.4 (-4.5;3.6)						
Diet	79.8 (12.1)	80.4 (13.7)	0.6 (-2.2;3.3)	0.75 (-3.42;4.93)	0.72	0.07			
Exercise	77.2 (13.0)	79.8 (12.4)	2.6 (0.1;5.0)	1.62 (-2.55;5.79)	0.44	0.15	0.87 (-2.37;4.11)	0.60	0.08
PCS (n = 214)									
Control	54.0 (5.1)	54.5 (6.8)	0.5 (-1.4;2.3)						
Diet	52.6 (7.2)	53.3 (6.5)	0.6 (-0.6;1.9)	-0.39 (-2.34;1.56)	0.70	0.08			
Exercise	53.2 (6.0)	54.5 (6.0)	1.3 (0.2;2.5)	0.52 (-1.42;2.46)	0.60	0.10	0.91 (-0.62;2.43)	0.24	0.18
MCS (n = 214)									
Control	53.7 (6.2)	53.2 (6.6)	-0.5 (-2.0;0.9)						
Diet	52.7 (8.0)	52.6 (8.2)	-0.1 (-2.0;1.7)	-0.11 (-2.46;2.23)	0.92	0.02			
Exercise	52.1 (8.2)	52.9 (6.8)	0.8 (-0.6;2.2)	0.49 (-1.86;2.84)	0.68	0.08	0.60 (-1.24;2.45)	0.52	0.10
Health change (n = 214)									
Control	53.8 (17.7)	52.6 (17.0)	-1.2 (-8.1;6.1)						
Diet	51.4 (17.2)	61.8 (20.5)	10.4 (5.9;14.8)	8.81 (1.55;16.1)	0.02	0.53			
Exercise	50.3 (12.9)	73.6 (19.1)	23.3 (18.7;27.9)	20.5 (13.2;27.7)	<0.01	1.18	11.7 (5.91;17.4)	<0.01	0.65

PCS: Physical Component Summary score (1–5). MCS: Mental Component Summary score (4–8). NOTE 1. As complete cases are presented, i.e., women who filled in a questionnaire both at baseline and follow-up, baseline scores may differ from the baseline scores as presented in Table 1. n = 214 (88.1%) (control group, n = 39; diet group, n = 87; exercise group, n = 88). Analyses were according to the intention-to-treat principle in all complete cases by linear regression with adjustment for the baseline SF-36 domain score. NOTE 2. The eight SF-36 domain scores range from 0 to 100, a higher score on the scale indicates a better health status. The score of the domain ‘*health change’* ranges from 0 to 100, a score of 50 means no change in perceived health, a score <50 denotes deterioration and >50 improvement. The summary scores PCS and MCS are sex-standardised and represent health scores of the Dutch population with a mean of 50, and a standard deviation (SD) of 10. For example, a PCS score of 60 indicates that physical health is improved with one SD in our study population compared to the general female population.

The effect sizes of differences in HRQoL between the study groups were small to medium for all SF-36 domains, except for *health change* which had medium to large effect sizes for all comparisons, with the largest effect size in the exercise group versus control of 1.18.

### Associations between change in weight or physical fitness and change in HRQoL in the total study population

Both weight loss and an increase in fitness level were significantly associated with an increase in *health change* score (Tables 3 and 4) (St-b = 0.4, 95% CI 0.3 to 0.5, and St-b = 0.3, 95%CI 0.1 to 0.4, respectively, in the unadjusted models (model 1)). After adjustment for covariates, these associations slightly attenuated but remained significant.

**Table 3:** Association between change in weight (weight loss) and change in HRQoL, regardless of study group.

	Crude Model 1*			Adjusted model 2†			Adjusted model 3‡		
	St-b§	95% CI	P-value	St-b	95% CI	P-value	St-b	95% CI	P-value
**PCS**	0.08	-0.05;0.22	0.24	0.14	-0.04;0.32	0.12	0.10	-0.08;0.28	0.27
**MCS**	0.07	-0.07;0.21	0.32	0.13	-0.04;0.32	0.12	0.09	-0.07;0.26	0.25
**Health change**	0.42	0.30;0.54	<0.001	0.29	0.13;0.45	<0.001	0.28	0.12;0.44	<0.001

PCS: Physical Component Score (1–5). MCS: Mental Component Score (4–8).

*Crude model 1 = Weight loss (i.e. Weight at baseline minus Weight at 16 weeks) = independent, (SF-36 Score at 16 weeks minus SF-36 Score at baseline) = dependent.

†Adjusted model 2 = model 1 adjusted for intervention group, age, education, baseline SF-36 score and baseline weight

‡Adjusted model 3 = model 1 adjusted for intervention group, age, education, baseline SF-36 score, baseline weight and change in VO_2peak_ (mL/min)

§St-b (with 95% confidence interval, 95%CI) is the regression coefficient from linear regression models that represents the effect on standard deviations (SD) change in HRQoL, per one SD change in weight. E.g., an St-b of 0.16 means that if weight loss increases by 1 SD, the mean SF-36 domain score increases by 0.16 SD.

**Table 4:** Association between change in fitness (VO_2peak_) and change in HRQoL, regardless of study group.

	Crude Model 1*			Adjusted model 2†			Adjusted model 3‡		
	St-b§	95% CI	P-value	β	95% CI	P-value	β	95% CI	P-value
**PCS**	0.002	-0.14;0.14	0.98	0.05	-0.10;0.19	0.54	0.05	-0.10;0.19	0.54
**MCS**	0.05	-0.08;0.19	0.44	0.11	-0.02;0.24	0.11	0.11	-0.02;0.24	0.11
**Health change**	0.26	0.12;0.40	<0.001	0.18	0.04;0.31	0.01	0.17	0.05;0.30	0.01

PCS: Physical Component Score (1–5). MCS: Mental Component Score (4–8).

*Crude model 1 = (Fitness at 16 weeks minus Fitness at baseline) = independent, (SF-36 Score at 16 weeks minus SF-36 Score at baseline) = dependent.

†Adjusted model 2 = model 1 adjusted for intervention group, age, education, baseline SF-36 score and baseline VO_2peak_ (per 10 mL/min)

‡Adjusted model 3 = model 1 adjusted for intervention group, age, education, baseline SF-36 score, baseline VO_2peak_ (per 10 mL/min) and change in weight.

§St-b (with 95% confidence interval, 95%CI) is the regression coefficient from linear regression models that represents the number of standard deviations (SD) change in HRQoL (dependent variable), per 1 SD change in fitness VO_2peak_, per 10 mL/min, independent variable). E.g., an St-b of 0.16 means that if fitness increases by 1 SD, the mean SF-36 domain score increases by 0.16 SD.

Although the coefficients of both change in weight and change in fitness were pointing in the hypothesised direction for the physical and mental component summary scores (i.e. weight loss and increased physical activity increase summary scores), the effects were small and not statistically significant.

## Discussion

We investigated the effects of comparable exercise- and diet-induced weight loss (6%-7%) on HRQoL, among overweight and inactive postmenopausal women. Only self-perceived health status improved in comparison to one year ago (referring to the period before study enrollment). The effect is medium to large, according to Cohen’s *d*, and statistically significant in both intervention groups versus control. Moreover, the improvement was significantly larger in the exercise group when compared to diet. Although most domains of HRQoL improved with diet and exercise, and improvements were on average larger in the exercise group, none of these improvements were statistically significant and effects were small to medium according to Cohen’s *d* effect size.

A meta-analysis by Warkentin et al. evaluated 53 RCTs wherein weight loss was induced by several modalities in different types of populations [8]. They concluded that weight loss may increase physical, but not mental health. However, the authors concluded that compelling evidence for the association between weight loss and HRQoL is still lacking as many studies, in line with our results, do not find significant effects of weight reduction [8].

We only observed an effect on self-perceived health (domain *health change)*. There is limited information on the psychometric properties of this single question. One study showed the item to be capable to better detect changes in general health than purely physical or mental aspects of health [25]. Another research concluded that this single question reflects SF-36 outcomes well at a group level [26].

In our research, all other domains of the SF-36 showed small to moderate and non-significant results. This might have been caused by including women with relatively high QOL scores at baseline, leaving little room for improvement. This ceiling effect may limit generalizability of the results, which is a limitation for the study. Intervention studies in older women with physical impairments show generally lower baseline HRQoL scores and weight loss results in better improvements [27,28]. Furthermore, despite its validation, the SF-36 is a generic HRQoL questionnaire which might not be specific enough to detect subtle changes in our study population.

The strengths of this study are the randomised controlled design and the relatively large study population. Furthermore, there was high adherence in both intervention groups to the different weight loss programmes. Moreover, the design enabled us to study effects of different intervention methods, because the amount of weight loss was comparable in both groups.

Our weight loss, though, was rather modest, but some trials found small but significant effects on HRQoL after modest weight loss in a population of healthy postmenopausal women [29–33].

Two large RCTs, the NEW trial [29] and a trial by Villareal et al. [32], investigated the individual and combined effects of a hypocaloric diet and/or exercise interventions on HRQoL during one year in healthy older women. In both trials improvements in HRQoL were observed in the intervention groups that concordantly lost body weight, that is, the diet-only and combined diet and exercise intervention group.

In the NEW trial, the combined diet and exercise intervention group showed improvements in five SF-36 domain scores, while the diet-only group only improved in two domains including vitality and mental health [29]. In contrast to our current study the NEW trial did not aim for a specific and equivalent weight loss across the intervention groups. This combined intervention group lost approximately -9 kg (-10.8%) body weight while the diet-only lost -7 kg (-8.5%). In the exercise only intervention group of the NEW trial (-2 kg, -2.4% weight loss) no effects were observed on HRQoL.

Villareal et al. reported on the effects of the interventions on the Physical and Mental Component Summary scores only. Both the diet and combined diet plus exercise groups achieved a weight loss of 9–10% and showed the same significant improvement in the PCS of the SF-36 [32]. No effect of either of the study interventions was seen on MCS. In the SHAPE-2 study we had a slightly smaller weight loss (6%-7%) and the duration of our intervention was much shorter (16 weeks compared to one year) which could also explain the different results.

Even though some studies showed that exercise even without concurrent weight loss may increase HRQoL [10–13], the above trials did not find effects in the exercise only group or additional effects of exercise when weight loss was reached [29,32].

In a non-randomised study among 298 overweight women, aged 50–75 years, it was tried to investigate the individual contribution of weight loss and change in physical fitness to change in HRQoL [30]. After a 6-month lifestyle treatment for obesity, approximately 9 kg body weight (9.5%) was lost and physical fitness (measured by a 6-minute walk test) increased by 5.5%. Weight loss appeared to contribute significantly to improvements in seven out of nine HRQoL domains, while physical fitness did not further improve HRQoL, not even in the domain *health change*. In our study, the effects of weight loss were slightly higher when compared to fitness, which suggestively supports the findings of Ross et al [30]. However, the difference between the influence of these parameters on HRQoL is rather small. Furthermore, for the domain *health change*, both parameters contributed significantly. We, therefore, conclude that both change in weight and fitness contribute to effects on HRQoL.

To conclude, our study showed that modest weight loss of 6%-7% resulted in a positive change in self-perceived health status in a population of healthy overweight and obese, inactive, postmenopausal women. This change was significantly larger when weight loss was achieved mainly by exercise compared with equivalent weight loss by diet alone, indicating an effect of exercise beyond weight loss. Furthermore, there was a tendency towards improvements in HRQoL in physical and mental SF-36 domains in both intervention groups, which were most pronounced in the exercise group.

## Supporting Information

S1 File SHAPE-2 trial study protocol. (DOC)

S2 File CONSORT checklist SHAPE-2 trial (DOC)
