# Supplementary material for: Quality of Life after Diet or Exercise-Induced Weight Loss in Overweight to Obese Postmenopausal Women: The SHAPE-2 Randomised Controlled Trial
Source: PLoS One. 2015 Jun 1;10(6):e0127520. doi: 10.1371/journal.pone.0127520 (PMC4452367; doi:10.1371/journal.pone.0127520)
Supplement: S1 File — (DOC) [file pone.0127520.s001.doc]

STUDY PROTOCOL

**SHAPE-2 Study:**

**Sex-Hormones And Physical Exercise**

**
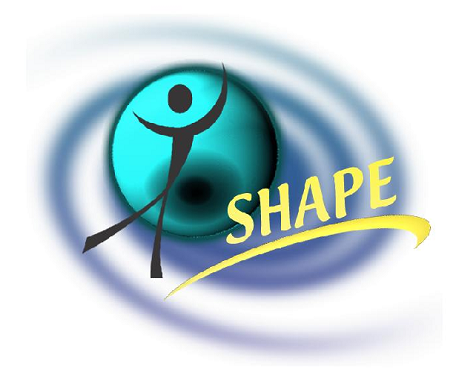
**

Drs. WAM van Gemert^1^, Dr. EM Monninkhof^1^, Prof. Dr. Ir. AJ Schuit^2^, Prof. Dr. PHM Peeters^1^

1. University Medical Center Utrecht, Julius Center for Health Sciences and Primary Care
2. Netherlands National Institute for Public Health and Environment (RIVM) department of Health prevention and policy.

**SHAPE-2 study: Sex-Hormones and Physical Exercise**

**The effects of equivalent weight loss with or without exercise training on breast cancer risk biomarkers in postmenopausal women: the SHAPE-2 study.**

**Dutch title: Het effect van afvallen met of zonder beweegprogramma op het borstkankerrisico van postmenopauzale vrouwen: de SHAPE-2 studie.**

| **Protocol ID** | **11-465** |
| --- | --- |
| **Short title** | **SHAPE-2 study** |
| **Version** | **1** |
| **Date** | **22-05-12** |
| **Coordinating investigator/project leader** | **E.M. Monninkhof, PhD**  **UMCU/JC. Universiteitsweg 100 Str. 6.131, 3508 GA Utrecht. tel 088-7559379**  **e.monninkhof@umcutrecht.nl**  **P.H.M. Peeters. MD, PhD**  **UMCU/JC. Universiteitsweg 100 Str. 6.131, 3508 GA Utrecht. tel 088-7559363.** [**p.h.m.peeters@umcutrecht.nl**](mailto:p.h.m.peeters@umcutrecht.nl)  **A.J. Schuit. Ir, PhD**  **RIVM. Center for Health Promotion and Policy, Postbus 1, 3720 BA Bilthoven.**  **Tel: 030-2742945** |
| **Principal investigator(s) (in Dutch: hoofdonderzoeker/uitvoerder)**  ***Multicenter research: per site*** | **UMCU UTRECHT:**  **E.M. Monninkhof, PhD**  **UMCU/JC. Universiteitsweg 100 Str. 6.131, 3508 GA Utrecht. tel 088-7559379**  **e.monninkhof@umcutrecht.nl**  **W. van Gemert, MD**  **UMCU/JC. Universiteitsweg 100 Str. 6.131, 3508 GA Utrecht. tel: 088-7568251**  **w.vangemert@umcutrecht.nl**  **MST ENSCHEDE:**  **J. van der Palen, PhD**  **Medisch Spectrum Twente, Haaksbergerstraat 55, 7500 KA, Enschede. Tel: 053-487 36 66**  **j.vanderpalen@mst.nl** |
|  |  |
| **Sponsor (in Dutch: verrichter/opdrachtgever)** | **UMCU** |
|  |  |
| **Independent physician(s)** | **UMCU UTRECHT:**  **M.R. Moman, MD PhD**  **UMCU, Department of radiology. Heidelberglaan 100, 3584CX Utrecht. Tel: 088-755 6689**  **m.r.moman@umcutrecht.nl**  **MST ENSCHEDE:**  **P.D.L.P.M. van der Valk, MD PhD**  **Medisch Spectrum Twente, department of pulmonology, Haaksbergerstraat 55, 7500 KA, Enschede. Tel: 053-487 26 10**  [**Onderzoeksbureau.Longgeneeskunde@ziekenhuis-mst.nl**](mailto:Onderzoeksbureau.Longgeneeskunde@ziekenhuis-mst.nl) |
|  |  |

**PROTOCOL SIGNATURE SHEET**

| **Name** | **Signature** | **Date** |
| --- | --- | --- |
| **K.G.M. Moons, MD, PhD** |  |  |
| **P.H.M. Peeters, MD, PhD** |  |  |
| **J. van der Palen, PhD** |  |  |
| **E.M. Monninkhof, PhD** |  |  |

**TABLE OF CONTENTS**

LIST OF ABBREVIATIONS AND RELEVANT DEFINITIONS [7](#__RefHeading___Toc310248352)

SUMMARY [8](#__RefHeading___Toc310248353)

1. INTRODUCTION AND RATIONALE [9](#__RefHeading___Toc310248354)

1.1 Introduction [9](#__RefHeading___Toc310248355)

1.2 Hypothesis to be tested [10](#__RefHeading___Toc310248356)

1.3 Sex hormones and postmenopausal breast cancer risk [10](#__RefHeading___Toc310248357)

1.4 The effects of physical activity on sex hormones [10](#__RefHeading___Toc310248358)

1.5 The effects of obesity and weight loss on sex hormone levels [11](#__RefHeading___Toc310248359)

1.6 Conclusions and rationale for the study [12](#__RefHeading___Toc310248360)

2. OBJECTIVES [13](#__RefHeading___Toc310248361)

2.1 Primary objectives [13](#__RefHeading___Toc310248362)

2.2 Secondary objectives [13](#__RefHeading___Toc310248363)

3. STUDY DESIGN [14](#__RefHeading___Toc310248364)

3.1 Run-in period [14](#__RefHeading___Toc310248365)

3.2 Diet-induced weight loss intervention (group D) [15](#__RefHeading___Toc310248366)

3.3 Combined exercise- plus diet-induced weight loss (Group E) [16](#__RefHeading___Toc310248367)

3.4 Waiting list control group (group C) [20](#__RefHeading___Toc310248368)

4. STUDY POPULATION [22](#__RefHeading___Toc310248369)

4.1 Population (base) [22](#__RefHeading___Toc310248370)

4.2 Inclusion criteria [22](#__RefHeading___Toc310248371)

4.3 Exclusion criteria [22](#__RefHeading___Toc310248372)

4.4 Sample size calculation [22](#__RefHeading___Toc310248373)

5. METHODS [24](#__RefHeading___Toc310248374)

5.1 Study parameters/endpoints [24](#__RefHeading___Toc310248375)

5.1.1 Main study parameter/endpoint [24](#__RefHeading___Toc310248376)

5.1.2 Secondary study parameters/endpoints [24](#__RefHeading___Toc310248377)

5.1.3 Other study parameters [24](#__RefHeading___Toc310248378)

5.2 Randomisation, blinding and treatment allocation [24](#__RefHeading___Toc310248379)

5.3 Study procedures/data collection [24](#__RefHeading___Toc310248380)

5.4 Withdrawal of individual subjects [26](#__RefHeading___Toc310248381)

6. SAFETY REPORTING [27](#__RefHeading___Toc310248382)

6.1 Section 10 WMO event [27](#__RefHeading___Toc310248383)

6.2 Adverse and serious adverse events [27](#__RefHeading___Toc310248384)

6.3 Follow-up of adverse events [27](#__RefHeading___Toc310248385)

7. STATISTICAL ANALYSIS [27](#__RefHeading___Toc310248386)

7.1 Descriptive statistics [27](#__RefHeading___Toc310248387)

7.2 Analysis [28](#__RefHeading___Toc310248388)

8. ETHICAL CONSIDERATIONS [28](#__RefHeading___Toc310248389)

8.1 Regulation statement [28](#__RefHeading___Toc310248390)

8.2 Recruitment and consent [28](#__RefHeading___Toc310248391)

8.3 Compensation for injury [29](#__RefHeading___Toc310248392)

8.4 Incentives [30](#__RefHeading___Toc310248393)

9. ADMINISTRATIVE ASPECTS AND PUBLICATION [30](#__RefHeading___Toc310248394)

9.1 Handling and storage of data and documents [30](#__RefHeading___Toc310248395)

9.2 Monitoring [30](#__RefHeading___Toc310248396)

9.3 Amendments [30](#__RefHeading___Toc310248397)

9.4 Annual progress report [30](#__RefHeading___Toc310248398)

9.5 End of study report [31](#__RefHeading___Toc310248399)

9.6 Public disclosure and publication policy [31](#__RefHeading___Toc310248400)

10. REFERENCES [32](#__RefHeading___Toc310248401)

# LIST OF ABBREVIATIONS AND RELEVANT DEFINITIONS

| **ABR** | **ABR form, General Assessment and Registration form, is the application form that is required for submission to the accredited Ethics Committee (In Dutch, ABR = Algemene Beoordeling en Registratie)** |
| --- | --- |
| **AE** | **Adverse Event** |
| **AR** | **Adverse Reaction** |
| **CA** | **Competent Authority** |
| **CCMO** | **Central Committee on Research Involving Human Subjects; in Dutch: Centrale Commissie Mensgebonden Onderzoek** |
| **CV** | **Curriculum Vitae** |
| **DSMB** | **Data Safety Monitoring Board** |
| **EU** | **European Union** |
| **EudraCT** | **European drug regulatory affairs Clinical Trials** |
| **GCP** | **Good Clinical Practice** |
| **IB** | **Investigator’s Brochure** |
| **IC** | **Informed Consent** |
| **IMP** | **Investigational Medicinal Product** |
| **IMPD** | **Investigational Medicinal Product Dossier** |
| **METC** | **Medical research ethics committee (MREC); in Dutch: medisch ethische toetsing commissie (METC)** |
| **(S)AE** | **(Serious) Adverse Event** |
| **SPC** | **Summary of Product Characteristics (in Dutch: officiële productinfomatie IB1-tekst)** |
| **Sponsor** | **The sponsor is the party that commissions the organisation or performance of the research, for example a pharmaceutical**  **company, academic hospital, scientific organisation or investigator. A party that provides funding for a study but does not commission it is not regarded as the sponsor, but referred to as a subsidising party.** |
| **SUSAR** | **Suspected Unexpected Serious Adverse Reaction** |
| **Wbp** | **Personal Data Protection Act (in Dutch: Wet Bescherming Persoonsgevens)** |
| **WMO** | **Medical Research Involving Human Subjects Act (in Dutch: Wet Medisch-wetenschappelijk Onderzoek met Mensen** |

# SUMMARY

**Rationale:** Postmenopausal women who are sedentary or overweight, have an increased breast cancer risk. It is suggested that these two factors mediate breast cancer risk mainly through sex hormone-related pathways. However, an inactive lifestyle and obesity are highly correlated and it is not clear which is most relevant to risk.

Literature shows that weight loss/ fat loss reduces postmenopausal sex hormone levels, but the question is if there is an additional beneficial effect on hormones of reaching this weight loss by physical activity instead of nutritional interventions.

We, therefore, propose to study the effect of weight loss mainly driven by exercise compared to equivalent weight loss due to calorie restriction only on sex hormones.

**Objective**: To examine the effects of equivalent weight loss with or without exercise training on breast cancer biomarkers in overweight, sedentary postmenopausal women

**Study design:** three-armed, single-blind, randomized controlled intervention study. Two hundred and fifty eligible postmenopausal women will first enter a run-in period of 5 weeks. During this period, all participants get an isocaloric diet adapted to the guidelines for healthy nutrition. After the run-in period, women are randomized into 3 groups: a diet- induced weight loss group (D); a combined exercise- plus diet-induced weight loss group (E); a control group (C) The aim for the women in both weight loss groups is to loose 5-6 kg of body weight. The intervention period lasts for 10-14 weeks. After the weight loss goal is achieved or after a maximum of 14 weeks intervention period, the maintenance period will start. In this period of 2-6 weeks, all participants will be weight-stabilized.

**Study population:** Healthy, overweight and obese (BMI 25-35 kg/m^2^), sedentary postmenopausal women (aged 50-69).

**Intervention:** Group D will follow an energy-restricted diet (-500 kcal/day). While group E will follow an exercise programme using 350 kCal/day combined with a (less strict) energy restricted diet (-250 kcal/day). The aim of both intervention groups is to loose 5-6 kg of body weight in 10-14 weeks.

**Main study parameters/endpoints:** The primary outcomes are serum estradiol (total, free), estrone, testosterone, sex hormone binding globulin. The secondary measurements include BMI, weight, waist- and hip-circumference, total body fat, abdominal fat (subcutaneous and visceral), fitness and blood pressure.

**Nature and extent of the burden and risks associated with participation, benefit and group relatedness:**

Benefit:

A benefit for the participants is that they will loose weight under supervision of a dietician and physiotherapist in a healthy way. This also applies to the control group since they will be offered an adapted weight loss program at the end of the study.

Burden:

-The weight loss programs are quite time consuming.

- Injuries due to exercise can occur, to minimize the risk the intensity of the exercise program will be gradually inclined during the study and supervised by a physiotherapist.

- Several measurements will be performed. At baseline, questionnaires about general health, medical history, reproductive history, exercise habits will be filled out. All participants will visit the hospital in their region for measurements twice including: history taking, physical examination, DEXA-scan, MRI-scan, maximal exercise capacity test and blood sampling. During the measurements, a haematoma can occur after blood sampling. Incidental findings can arise in the different measurements, which will be reported to participants. During the maximal capacity exercise test (clinically occult) pre-existing heart problems can be exposed. To reduce the risk of complications due to ischemia the participant will be screened for ischemic heart disease by ECG monitoring during the exercise test. Emergency equipment and personnel trained to deliver appropriate emergency care will be available.

# 1. INTRODUCTION AND RATIONALE

1.1. Introduction

1.2. Hypothesis to be tested

1.3. Sex hormones and postmenopausal breast cancer risk

1.4. The effects of physical activity on sex hormones

1.5. The effects of obesity and weight loss on sex hormone levels

1.6. Conclusions and rationale for the study

## Introduction

Breast cancer is a major public health problem: in the Netherlands, every year about 13.000 new cases occur and 3.180 women die from breast cancer. Most of the established risk factors for postmenopausal breast cancer are not easily amenable to intervention. In contrast, physical inactivity and obesity are modifiable risk factors and it is estimated that these two factors together may account for one third of all breast cancer cases^1^. A review of 19 cohort- and 29 case-control studies showed strong evidence for an inverse association between physical activity and postmenopausal breast cancer, with risk reductions ranging from 20-80%^2^. The biological mechanisms by which physical activity influences breast cancer risk is thought to be mainly through hormone-related pathways, i.e. sex steroid hormones and metabolic hormones (e.g. insulin)^3^.

In addition, many studies show that breast cancer risk is increased in obese postmenopausal women^4,5^. In a recent meta-analysis^6^, it was estimated that per 5 kg/m^2^ increase in body mass index (BMI = weight (kg) / [length (m)] ^2^), the risk of postmenopausal breast cancer increased by 12%. The increased breast cancer risk among postmenopausal women with a high BMI may largely be the result of associated increase in estrogens^7^. Thus far, there has been limited evidence from observational studies that weight loss after menopause reduces breast cancer risk^1, 8-11^. One of the reasons for this limited evidence is that in observational studies, it is generally impossible to distinguish healthy persons who intentionally loose weight from those who loose weight due to presence of early cancer or other chronic diseases (unintentional weight loss)^1^.

So, based on observational studies there is promising evidence that regular exercise is associated with decreased risk of postmenopausal breast cancer and that obesity is related with an increased risk. However, the effects of weight loss and the interplay between weight loss and physical activity on breast cancer risk are not fully clear. Intervention studies can provide insight in the biological mechanisms by which different behaviours influences breast cancer risk in postmenopausal women. We conducted an exercise-intervention study that suggested interplay between fat loss and physical activity, in accordance with an American trial. Both trials observed that physical activity influences postmenopausal hormone levels mainly when concordant loss of body fat is achieved.^12-14^ Our study also confirmed that fat loss is clearly associated with changes in postmenopausal sex hormone levels. On the other hand, a recently published Canadian exercise intervention study in postmenopausal women suggests an effect of exercise on sex hormones independent of changes in adiposity^15^, although the average weight reduction was higher in the intervention compared to the control group. But adjusting for weight changes still revealed beneficial effects of the activity intervention.

The following questions remain unanswered: When it comes to sex-hormone levels, does it make a difference whether weight loss is achieved through dietary interventions or through physical exercise? So does physical activity lower breast cancer biomarkers only through accompanied weight/fat loss? Or is there an additional hormone lowering effect of physical activity even if weight loss by physical activity is comparable to the loss achieved by a diet?

From recent literature, we know that achieving substantial weight loss ( 5-10% ) only by exercising in untrained and obese women is a long term process and a goal hard to attain.^16, 17^ Compensatory mechanisms both physically as mentally and behavioural will withhold the person from loosing weight adequately.^18, 19^ We, therefore, decided to study the effect of exercise in combination with a slight energy deficit.

The aim of this study is to provide insight into the effect of weight loss mainly driven by exercise compared to equivalent weight loss due to nutritional calorie restriction only on breast cancer biomarkers (sex hormones) in overweight, sedentary postmenopausal women. Furthermore, we are specifically interested whether weight loss mainly due to physical exercise induces greater amounts of fat loss (total and abdominal) and subsequently results in more favourable effects on these hormones compared to equivalent diet-induced weight loss.

## Hypothesis to be tested

We hypothesize that weight loss mainly driven by physical exercise induces a larger decrease in serum estradiol levels compared to equivalent diet-only induced weight loss and control.

To underpin this hypothesis, the association between sex hormones and postmenopausal breast cancer risk (3), the effect of physical activity and obesity on these hormones (4, 5) will be discussed in the next paragraphs.

## Sex hormones and postmenopausal breast cancer risk

The evidence that estrogens contribute to breast cancer risk is strong and widely accepted^20^ based on numerous observational studies. We conducted a large nested case-control study in the European Prospective Investigation into Cancer and Nutrition (EPIC)^21^. Levels of estrogens and androgens were measured in prediagnostic serum samples of 677 postmenopausal women who subsequently developed breast cancer and 1309 matched control subjects. For all estrogens, elevated serum levels were associated with a 2 fold increased breast cancer risk.

Another re-analysis of nine prospective studies also showed that a relatively high level of endogenous estrogens in postmenopausal women is associated with a 2-3 fold increased risk of breast cancer^22^. Postmenopausal women with elevated levels of androgens or dehydroepiandrosterone also showed increased risk of developing breast cancer, even after adjustment for estrogens^21-23^.

## The effects of physical activity on sex hormones

In many cross-sectional observational studies, a low level of physical activity has been associated with higher serum concentrations of estradiol, estrone and androgens in postmenopausal women^24-30^ but not in all^31, 32^. This might be partly explained by physical activity preventing becoming overweight since after menopause the principal source of estrogen production, peripheral aromatization of androstenedione is fat tissue^33-35^. Compared with normal-weight postmenopausal women, obese postmenopausal women have higher blood concentration of estrogens and androgens, and lower concentration of sex hormone binding globulin (SHBG)^7, 25, 36, 37^. Regular exercise represents an approach to regulate energy balance and to prevent the accumulation of adipose reserves and consequently influence the production of estrogens. Prevention of overweight, however, might not be the only pathway as most studies still observe an effect of physical activity on hormone levels after adjustment for BMI^25-29^. Physical activity might also influence postmenopausal sex hormones by increasing levels of sex hormone binding globulin (SHBG), resulting in lower amounts of unbound (free), active estrogens and androgens in circulation^26, 27, 38,30^.

So far, three trials were published assessing the effects of year-long exercise interventions on postmenopausal sex hormone levels^12-15^. The study of McTiernan et al.^12, 13^ investigated the effect of moderate-intensity exercise in 173 sedentary, overweight postmenopausal US women. after 3 months, significant declines in estrone (-4%), estradiol (-8%) and free estradiol (-8%) were observed compared to no change or increased levels in control women, although after 12 months the differences were no longer statistically significant. In women who lost >2% of body fat, however, significant decreases in serum estrogens and androgens were observed after 12 months. In this subgroup, serum estradiol and testosterone concentrations fell by 13.7% and 10.1 % between baseline and 12 months in exercisers compared with an increase of 4.7% and a decrease of 1.6% in controls, respectively.

The second intervention study was done by our group. In the SHAPE study, we assessed the effect of a moderate-to-vigorous intense exercise programme on sex hormone levels in 189 postmenopausal sedentary women (KWFno: UU 2003-2793)^14^.We also did not find an overall intervention effect on sex hormone levels, but again in women who lost >2% body fat androgen levels (testosterone and androstenedione) were on average, lower in the intervention group compared to the control group (8% and 10%, respectively).

The third trial (ALPHA trial (Alberta Physical Activity and Breast Cancer Prevention Trial)) assessed a year-long aerobic exercise trial among 320 postmenopausal Canadian women. This study resulted in significant reductions in estradiol and free estradiol by 13% compared with reductions of 3% and 4% in the control group, respectively. Additionally, SHBG levels increased significantly in the intervention group compared to controls. Adjustment for weight change during the study attenuated the effect on SHBG but not on (free) estradiol levels. The difference in overall effect between the studies might be caused by the effects of the intervention on weight change: -1.4 kg in McTiernan study, -0.5 kg in SHAPE trial and -2.3 in the ALPHA trial. On the other hand, the ALPHA study suggests an effect of exercise on estradiol independent of changes in adiposity by analysing the intervention effect after adjustment for weight change.

## The effects of obesity and weight loss on sex hormone levels

Many observational studies showed cross-sectional associations between BMI and estrogen levels in postmenopausal women^32, 39-42^. Obese postmenopausal women have up to 2-fold higher serum concentrations of estradiol than lean postmenopausal women^35^. The association between BMI and androgens is less clear, i.e. cross-sectional analyses produced conflicting results^26, 40, 42, 43^. Longitudinal data of the SHAPE study confirmed that loss of total body fat was significantly associated with declines in postmenopausal estrogen levels. Although not significant, a similar trend was observed for the androgens^14^.

There are also a number of uncontrolled intervention studies on the effects of weight loss on sex steroid hormones. A study in obese postmenopausal women showed that weight loss lowers circulating estrogens 6-12 month after participation in a weight reduction programme^44^. Since this study was uncontrolled, it is not clear which part of the reduction in estrogen levels is an effect of aging. In a review of Wu et al.^45^, the effects of reduction in dietary fat on estradiol were summarised. Five uncontrolled studies among postmenopausal women showed a 23 percent reduction in serum estradiol levels. In these studies, it is difficult to unravel the effects of weight loss or dietary fat reduction. The Diet and Androgens (DIANA) randomized trial ^46^, studied the effect of a multifactorial dietary intervention (e.g. low in animal fat, high in phytoestrogens) on sex hormones over 4.5 months in 104 Italian postmenopausal women with high serum testosterone levels. The dietary intervention group showed significant decreases in body weight (-4.06 kg vs. -0.54 kg in the control group) and testosterone levels (-20% and -7%), while SHBG levels (25% vs. 4%) and estradiol levels increased (-18%; -5.5%; although not significant).

## Conclusions and rationale for the study

Observational studies provide strong evidence that physical inactivity and overweight increase breast cancer risk in postmenopausal women. In contrast to most other risk factors for postmenopausal breast cancer, physical inactivity and overweight offer a potential basis for primary prevention since they are modifiable. Evidence, mostly from observational studies, is strong that physical inactivity and overweight mediate breast cancer risk mainly through sex hormone-related pathways. The results of dietary intervention studies (mostly uncontrolled) suggest that diet-induced weight loss reduces postmenopausal sex hormone levels.

The question remains if the beneficial effect of physical activity on breast cancer risk is fully explained by the accompanied weight loss. Information comes from three exercise intervention studies (RCTs) of which one conducted by ourselves. Two out of these suggest that exercise indeed influences hormone levels mainly when concordant loss of body fat is achieved. This implicates that particularly body fat is involved in the biological pathway between physical activity and hormone levels. However, since in both studies fat loss/weight loss was achieved by exercise it remains unclear if the effect would have been the same if fat loss/weight loss was achieved by calorie restriction. Physical activity may have an additional beneficial effect. Moreover, the third exercise intervention study suggests an effect of exercise on sex hormones independent of changes in adiposity.

We, therefore, propose to study the effect of weight loss mainly driven by exercise compared to equivalent weight loss due to a calorie restricted diet only on sex hormone levels.

Furthermore, we are specifically interested whether weight loss mainly driven by physical exercise induces greater amounts of fat loss (total and abdominal) and subsequently results in more favourable effects on these hormones compared to equivalent diet-induced weight loss.

# OBJECTIVES

## Primary objectives

To examine the effects of equivalent weight loss with and without exercise on breast cancer biomarkers (endogenous sex hormones) in overweight, sedentary postmenopausal women.

## Secondary objectives

To examine the effects on body(fat) composition and whether this mediates potential changes in endogenous sex hormones

# **STUDY DESIGN**

The SHAPE-2 study is designed as a single blind RCT with three study arms. The outcome assessors will be blinded for treatment assignment as much as possible. After a run-in period of 5 weeks, eligible women (n=250) will be randomised into a diet-induced weight loss group (D), a combined exercise- plus diet-induced weight loss group (E), or a waiting list control group (C). This intervention period will take 10-14 weeks. And will be followed by a weight maintenance period of 2-6 weeks, so that the total study duration is 21 weeks.

## Run-in period

During the 5-week run-in phase, all participants will follow a baseline diet. This diet is based on their regular diet but balanced to their assessed energy requirements. Individual energy requirements will be assessed by combining the results from 1) their reported habitual daily intake (dietary history) and weight history; and 2) using the adapted Harris&Benedict formula^48^ (for estimating resting energy expenditure) multiplied by an individualized factor for their habitual physical activity level (estimated by a questionnaire). The estimate of energy requirements will be evaluated (and adapted if necessary) based on body weight measurements during the run-in phase. The planned macronutrient profiles will be according to the Dutch Nutritional Guidelines: 50-60% carbohydrate, 15-20%protein and 20-35% fat^49, 50^. All participants will be provided with healthy diet recommendations according the Dutch guidelines:

- 150 to 200 grams of veg­eta­bles and 200 grams of fruit a day
- 30 to 40 grams a day of dietary fibre, es­pe­cial­ly from sources such as fruit, veg­eta­bles and whole-grain cereal prod­ucts
- Two por­tions of fish a week (one portion oily fish)
- Sat­u­rat­ed fatty acid con­sump­tion <10% of energy intake and mono trans-fat­ty acid con­sump­tion <1% of energy intake
- Limited con­sump­tion of foods and bev­er­ages that contain easily fer­mentable sugars and drinks that are high in food acids, to seven oc­ca­sions a day (in­clud­ing main meals)
- Limited con­sump­tion of table salt to 6 grams a day
- Alcohol intake should be limited to one Dutch units a day
- Special attention will be paid to calcium and vitamin D intake according to age-specific recommendations, since weight loss without exercise in may also result into loss of bone mass^51, 52^.

Dietary counselling during the run-in phase will consist of an individual intake interview by the dietician. During this start interview, the habitual dietary pattern is recorded using a dietary history and a dietary plan is discussed for weight maintenance. The dietician will contact the participants two times by telephone to evaluate body weight, dietary compliance and the accuracy of the prescribed energy intake and discuss diet adaptations if necessary

Body weight will be self-monitored by the participants weekly and noted down in an online diary. For measuring protocol adherence, dietary intake will be measured during the study in all study arms. For this purpose, participants will be asked to complete a food-record (as described below in paragraph 3.2) once during the run-in, intervention and maintenance period. See Figure 1 for the exact time schedule.

For measuring baseline physical activity level all participants will wear the ActiGraph (as described in paragraph, an activity monitor, in 7 consecutive days during the run-in period.

## Diet-induced weight loss intervention (group D)

Women randomised to diet only group will be prescribed a diet creating a mean energy deficit of 500 kcal/day during a weight reduction period of 10-14 weeks. This is assumed to produce a weight loss of 5-6 kg in total (approximately 0.5 kg per week). Diet composition and recommendations will be comparable to the baseline diet.

The weight reduction period, will be followed by a weight maintenance period of 2-6 weeks in which energy intake and energy expenditure will be balanced. The weight maintenance period starts as soon as the individual weight reduction goal is obtained (total weight loss of 5-6 kg), or in study week 19. From this time point until end of study, energy intake should meet the individual energy requirements for weight maintenance.

*Dietary counselling during the weight reduction intervention phase (see fig 1) will consist of:*

- Two individual 30 minutes individual sessions with a dietician. During the first session (week 6), individualised instructions are given on how to create the daily energy deficit of 500 kcal and perceived individual pitfalls will be discussed. The second session will take place at the end of the weight reduction phase in order to instruct the participant on how to maintain the reduced body weight.
- Five group sessions: In these one-hour sessions, participants in this treatment arm will be trained for self-management of body weight reduction and maintenance at the long run.
- Seven phone calls: the dietician will contact the participants by phone to evaluate body weight, motivate them and discuss diet adaptations if necessary.
- Completion of a food-record once during the intervention period. Participants will be instructed by the dietitian to record their daily food intake using a food-diary for 3 consecutive days (2 weekdays and 1 weekend day) De dietitian will provide feedback on energy and nutrient content of the reported intake

*Background principles and structure of the weight loss intervention:*

We use several theoretical models^53-55^ putting different emphasis on the key psychological processes of behavioral change. In accordance with these models, the following methods^56-59^ will be used to modify woman’s behavior depending on her individual characteristics and preferences:

- Self-monitoring of body weight and energy intake
- Goal-setting: participants will be instructed to set specific (i.e. quantifiable) behavioral goals for the timeframe until the next session. The goals should be realistic, yet moderately challenging.
- Stimulus control: participants must identify stimuli and high risk-situations. They should develop their own controlling strategies, such as learning to shop carefully for healthy food, keeping high-calorie foods out of the house, limiting the times and places of eating, and consciously avoiding situations in which overeating occurs.
- Problem solving: participants should identify and self-correct problem areas related to their eating
- Relapse prevention: several skills to overcome setbacks and to cope with problems will be taught.

*Mode of delivery:*

The weight loss intervention is delivered by dieticians experienced in overweight treatment. Individual and group sessions are planned. Group sessions are supposed to provide a combination of social support and a healthy dose of competition. Group treatment is also more cost-effective than individual care^60^. Furthermore, there is evidence that group support might produce greater weight loss effects than individual counselling alone^61^. A family member or other support person may be invited to attend any or all sessions to increase social support^62, 63^.

*Intensity and duration of contacts:*

Frequent contact between participants and team members is proven to be a relevant success factor for weight reduction^64^. In most studies^65^ lifestyle modification training is provided weekly for an initial period of 16 weeks. In our programme, the contact frequency will be quite similar (see Figure 1). The dietary intervention will consist of 2 one-hour individual sessions, 5 one-hour group sessions and 7 phone calls. A recently published large randomised controlled trial in overweight adults showed that with this kind of dietary intervention relevant weight loss can be achieved. This study found an average weight loss of 7% of initial weight after six months^66^.

*Group sessions:*

Group sessions will be conducted by a dietician using a structured curriculum in groups of 10 women. The nutritional education will include information on the importance of regular meal takings, the need for breakfast, the influence of snacking and grazing, a closer look at portion sizes and food labels, and the energy density of several food groups. Social support will be stimulated during the sessions using group discussion and reinforcement techniques. At each session, women review each others weight history and help each other generating strategies to cope with problems identified. Also personal feedback will be given by the dietician.

Lecture time will be kept to a minimum in favour of participants asking questions or discussing their pitfalls or achievements. Visits conclude with discussion of homework assignments for the coming weeks and the instruction not to change their habitual physical activity level.

*Protocol adherence:*

Adherence to the protocol will be determined during each phone call and individual/group meeting by examination of body weight and by energy intake estimated by the food-record and the dietician. Participants, whose weekly weight loss do not meet or exceeds the 0.5 kg/week goal for 3 consecutive weeks, will receive extra coaching and/or their diet will be adapted.

## Combined exercise- plus diet-induced weight loss (Group E)

Participants randomised to the combined exercise- plus diet-induced weight loss group will be enrolled in a 14 week structured exercise programme. Thereby, they follow an energy-restricted diet (which is less strict than for the diet-induced weight loss group). Goal of this combined programme is to produce a weight loss of 5-6 kg in total (approximately 0.5 kg per week) and to increase fitness. The weight reduction period, will be followed by a weight maintenance period of 2-6 weeks in which energy intake and energy expenditure will be balanced. The weight maintenance period starts as soon as the individual weight reduction goal is obtained (total weight loss of 5-6 kg), or in study week 19. Participants entering the maintenance period before week 19 will complete their 14 week exercise programme and can compensate correlated energy expenditure by increased caloric intake. From start of the maintenance period until end of study, energy intake should meet the individual energy requirements for weight maintenance.

Exercise Intervention

The 14 week exercise intervention programme creates an average energy expenditure of approximately 350 kCal/day. It consists of 2 one-hour supervised fitness group sessions at a physiotherapist centre. Plus 2 hours/week of individual home-exercise Nordic Walking. The training programme is a combination of high intensity endurance training and strength training. High intensity endurance training results in higher absolute fat oxidation rates than training of moderate intensity^67^. Knechtle and coworkers found that women’s fat oxidation rate was higher when cycling or running at 75% VO_2_peak than at 65% or 55% VO_2_peak. We choose for a combination with strength training since strength training is important for postmenopausal women to preserve and increase muscle mass and strength and to preserve bone mineral density^68^. Moreover, the advantages of strength training may have greater implications than initially proposed with respect to decreasing percentage body fat and sustaining fat free mass^69^. In addition, strength training might be helpful in combating the decrease in basal metabolic rate during the period of negative energy balance.

*Group Exercise*

Twice a week the participants of the combined exercise- plus diet-induced weight loss group will gather for a supervised exercise session for 60 minutes. The groups consist of 5-6 women. A physiotherapist who is extensively trained for the study will facilitate the standardised group sessions. Classes will start with a warming up. Then the training is continued with moderate-to-vigorous level of aerobic exercise (circuit training) on 60-90% of the peak heart rate (60-84% of VO2peak). The percentage of peak heart rate on which participants will train will increase gradually during the study programme (see table 1). The endurance training in week 9-14 will be a high-intensity interval training, with 30 seconds of intensive exercise and 60 seconds of active rest.  Exercise intensity was based on the guidelines of the American College of Sports Medicine, adapted for older woman^70^. Peak heart rate of each participant will be measured during a maximal exercise test. The participants will wear heart rate monitors to control their training intensity. They will also receive a badge with their target heart rates for a range of training intensities. The intensity and duration of the endurance training will be gradually inclined in the first weeks. Each training session will end with standardised strength exercises of the major muscle groups (see Tables 3a and 3b for an example of exercises; the type of exercise for a specific muscle group might be adapted to the facilities of the physiotherapist centre) and 5 minutes cooling down. The first week will be a run-in period for the strength exercises in which the technique of the exercises will be extensively instructed and practiced. Furthermore, in the first week the 20 repetition maximum (RM) (i.e. the maximum load that a muscle group can perform 20 repetitions of an exercise before becoming fatigued) will be determined for the different exercises (except for the abdominal exercises). This 20 RM will be the starting point for the strength training programme. To secure sufficient training load throughout the training programme, the 20 RM will be repeated at week 4; and a 15 RM will be determined at week 8 and 12.

Table 1. Content and composition of the group sessions

|  | **Time (min)** | **Intensity (% HF max for endurance part)** | | | | | |
| --- | --- | --- | --- | --- | --- | --- | --- |
|  |  | Phase 1 | | Phase 2 | | Phase 3 | |
| *Part of the session* |  | Wk 1-3 | | Wk 4-8 | | Wk 9-14 | |
| **Warming-up** | 5-10 | 60-65% | | 60-65% | | 60-65% | |
| **Endurance training** | 20-25 | 60-70% | | 70-80%  (5-10 min 80-90%) | | 70-90%  (high-intensity interval training; see table 2) | |
| **Strength exercises** | 25 | Increasing intensity and decreasing number of repetitions (see  Table 3a-b) | | | | | |
| **Cooling down** | 5 |  |  | |  | |  |

Table 2. High intensity endurance interval training during phase 3

| **Week** | Intensity | **Repetitions** |
| --- | --- | --- |
| 9 | 70% | 10 |
| 10 | 75% | 10 |
| 11 | 80% | 10 |
| 12 | 80% | 15 |
| 13 | 85% | 15 |
| 14 | 90% | 15 |

Table 3a. Strength exercises week 2-8

| **Muscle Group** | Exercise | **Intensity** |
| --- | --- | --- |
| Back | Rowing | 1x 20-25 reps |
| Chest | Bench press | 1x 20-25 reps |
| Legs (hips, glutes and thighs) | Squat | 1x 20-25 reps |
| Shoulder | Shoulder press | 1x 20-25 reps |
| Arms (biceps) | Bicepscurl | 1x 20-25 reps |
| Legs (hips, glutes and thighs) | Lunges | 1x 20-25 reps |
| Legs (calfs) | Calf-raises | 1x 20-25 reps |
| Arms (triceps) | Triceps extension | 1x 20-25 reps |
| Abdominal | Crunch | 1x 30-40 reps |

Table 3b. Strength exercises week 9-14

| **Muscle Group** | Exercise | **Intensity** |
| --- | --- | --- |
| Back | Rowing | 2x 15-20 reps |
| Chest | Bench press | 2x 15-20 reps |
| Legs (hips, glutes and thighs) | Squat | 2x 20-25 reps |
| Shoulder | Shoulder press | 2x 10-15 reps |
| Arms (biceps) | Bicepscurl | 2x 20-25 reps |
| Arms (triceps) | Triceps extension | 2x 20-25 reps |
| Abdominal | Crunch Hoover | 2x 30-50 reps |

The energy requirements of a group exercise session is based on the compendium of physical activities of Ainsworth et al^71, 72^.

In this compendium average metabolic equivalent (MET) rates are given. Since we have a specific study group of overweight and obese, sedentary postmenopausal women we calculated energy expenditure with the help of corrected MET’s specific for this population. Corrected MET’s can be calculated with the use of the Harris-Benedict formula.^73, 74^

We calculated a correction factor of 1.376

Based on the baseline characteristics of our former conducted comparable study^75^ with a subanalysis of women with a BMI >25 kg/m^2^ , for the SHAPE-2 study we assumed an average weight of 78 kg, length of 1.65 m and age of 58 years. This gives us a correction factor 1.376 for the standard METs resulting in MET rates of 11 METs for the aerobic training (8*1.376) and 8.3 METs for the strength training (6*1.376). This gives a total energy expenditure of 750 kCal per session (11*0.5*78 + 8.3*0.5*78).

The physiotherapist registers the attendance of the subjects. The study coordinator will perform several monitoring visits per exercise group.

Previous experience showed that group exercises are preferred in this age group and are better adhered to in the short and long term^76^(see also Preliminary results). For feasibility reasons, individual home-based exercise is also included.

*Individual Exercise*

The home-based exercise programme comprised Nordic Walking at 75-85% of the maximum heart rate for two times 60 minutes per week. Energy requirements were assumed based on calculated corrected MET’s from Ainsworth.^73, 74^ It is comparible to study outcomes of Figard-Fabre et al.^77^ when a higher heart rate of 130/min is assumed for our training programme. For an average woman in our study one 60-minute session of Nordic Walking will comprise an energy expenditure of 515 kCal (6.6*1*78).

Women will receive Nordic Walking poles. Nordic Walking technique will be demonstrated and practised during a group exercise session. To increase the compliance of this individual programme, women will be stimulated to walk with other participants (walking buddy). And supervised group sessions by a sports instructor will be organized. Women have to record the intensity and duration of their weekly Nordic Walk session in an exercise log. Furthermore, women will be asked to wear an activity monitor during the individual exercise sessions: the Actigraph^78^ ([www.theactigraph.com](http://www.theactigraph.com/)). The physiotherapists will use the Actigraph to check duration, frequency and calorie expenditure of the individual training sessions. For women who do not appreciate Nordic Walking, we will design an alternative programme with the same energy expenditure (e.g. cycling; swimming etc).

In conclusion, the exerciseprogramme, containing 2 hours of fitness training and 2 hours of Nordic walking, gives a total energy expenditure of 2530 kCal/week (2*750 + 2*515) for a modal participant. Resulting in an average energy expenditure of 360 kCal/day.

Procedures to enhance adherence to the exercise programme are: (1) use of group exercise sessions in combination with a simple individual programme, (2) personal feedback of the physiotherapists, (3) use of exercise logs and an activity monitor (4) stimulate women to find a walking buddy for the individual part, and (5) newsletters.

Diet Intervention

Next to the exercise programme, women enrolled in the combined exercise- plus diet-induced weight loss group will be prescribed an energy-restricted diet creating an extra energy deficit of 250 kcal/day during the weight reduction period of 10-14 weeks. Diet composition and recommendations will be comparable to the baseline diet.

The diet intervention consist of two individual 30 minutes individual sessions with a dietician, 7 telephonic contacts and completion of a food-record. Purpose and content of these contacts are similar as for the diet-group, described in paragraph 3.2.

Participants, whose weekly weight loss does not meet or exceeds the 0.5 kg/week goal for 3 consecutive weeks, will receive extra coaching and/or their programme will be adapted. Participants, who reach the individual weight reduction goal (weight loss of 5-6 kg) before week 14, will enter the weight maintenance period in which energy intake and energy expenditure will be balanced (i.e. they keep exercising but their diet will be adapted).

## Waiting list control group (group C)

Participants in the waiting list control are requested to retain body weight according to the isocaloric diet and to maintain their habitual exercise pattern. We will give very clear recommendations on this throughout the study. Potential changes in weight, exercise and eating pattern will be monitored by four phone calls by the researchers or dietician (See figure 1). After completion of the 5 month study period, the control participants will be provided with all study materials of the diet and exercise programme. Additionally, we will offer them four dietary weight loss group sessions and three exercise group sessions (including one exercise education session).

**Figure 1: Time Schedule**

|  | *Run-in* | | | | | | *Intervention* | | | | | | | | | | | | | | *Maintenance^*^* | |
| --- | --- | --- | --- | --- | --- | --- | --- | --- | --- | --- | --- | --- | --- | --- | --- | --- | --- | --- | --- | --- | --- | --- |
| *Week* | 0 | 1 | 2 | 3 | 4 | 5 | 6 | 7 | 8 | 9 | 10 | 11 | 12 | 13 | 14 | 15 | 16 | 17 | 18 | 19 | 20 | 21 |
| **DIETARY COUNCELLING** |  |  |  |  |  |  |  |  |  |  |  |  |  |  |  |  |  |  |  |  |  |  |
| Face to face instruction baseline diet | **X** |  |  |  |  |  |  |  |  |  |  |  |  |  |  |  |  |  |  |  |  |  |
| Face to face instruction energy restricted diet |  |  |  |  |  |  | **DE** |  |  |  |  |  |  |  |  |  |  |  |  |  |  |  |
| Face to face instruction maintenance diet |  |  |  |  |  |  |  |  |  |  |  |  |  |  |  |  | **---------- DE ---------** | | | |  |  |
| Group sessions for education and motivation |  |  |  |  |  |  |  | **D** |  | **D** |  | **D** |  |  |  | **D** |  |  |  | **D** |  |  |
| Individual monitoring, motivation and education by telephone |  |  | **X** |  | **X** |  | **C** |  | **DE** |  | **X** |  | **DE** |  | **DE** |  | **DE** |  | **DE** |  | **X** |  |
| **DIETARY MEASUREMENT** |  |  |  |  |  |  |  |  |  |  |  |  |  |  |  |  |  |  |  |  |  |  |
| Dietary history intake interview | **X** |  |  |  |  |  |  |  |  |  |  |  |  |  |  |  |  |  |  |  |  |  |
| 3-consecutive-day food-record |  |  | **X** |  |  |  |  |  |  | **X** |  |  |  |  |  |  |  |  |  |  | **X** |  |
| **OTHER MEASUREMENTS** |  |  |  |  |  |  |  |  |  |  |  |  |  |  |  |  |  |  |  |  |  |  |
| Weight self-monitoring | **X** | **X** | **X** | **X** | **X** | **X** | **DE** | **DE** | **DE** | **DE** | **X** | **DE** | **DE** | **DE** | **DE** | **DE** | **DE** | **DE** | **DE** | **DE** | **X** | **X** |
| Measurements e.g MRI, blood |  |  |  |  |  | **X** |  |  |  |  |  |  |  |  |  |  |  |  |  |  |  | **X** |

**X= all study groups**

**D = Diet-induced weight loss group only**

**DE = Diet-induced weight loss group and combined Exercise- plus diet-induced weight loss group**

**C= Control group**

* The weight maintenance period starts from week 10 as soon as the weight reduction goal of 5-6 kg is obtained, and at least in week 19.

# STUDY POPULATION

## Population (base)

Women will be recruited through a random selection out of the female population aging 50-69 years in the surroundings of Utrecht and Enschede. For their contact details, we will use the community register (GBA) of Utrecht and Enschede and the UMCU register with potential candidates for scientific research (POKA).

It is likely that enough women can be recruited from this random selection since this age group is mainly postmenopausal and in the Netherlands 55% of these women is overweight ^79^. Furthermore the participants will be recruited in two separate areas which will enlarge the number of possible candidates.

## Inclusion criteria

- Female

- 50-69 years of age

- Postmenopausal (>12 months after last menses)

- BMI 25-35 kg/m^2^

- Sedentary (<2 hours per week of at least moderately intensive activities (>4 MET))

- Willing to be randomly assigned to one of the three study arms

- Informed consent to participate in all screening and study activities.

## Exclusion criteria

- Presently using sex-hormones

- Maintenance use of corticosteroids

- Suffering breast cancer (or in medical history).

- Suffering other types of cancer now or in the past 5 years, except for non-melanoma skin cancers

- Suffering diabetes mellitus (type 1 and 2) or other endocrine related diseases

- Smoking

- Alcohol or drug abuse

- Any disorder that might impede participation in the exercise programme

- Following, or the intention to follow, a structured weight loss programme elsewhere

- In the investigator’s opinion, subject lacks the ability or motivation to fulfil the study programme completely and/or successfully in either one of the three arms

## Sample size calculation

Sample size calculations are based on the effect of the interventions on serum estradiol levels. The following comparisons will be made:

1) diet-induced weight loss versus combined exercise- plus diet-induced weight loss

2) diet-induced weight loss versus control

3) combined exercise- plus diet-induced weight loss versus control

The following assumptions are based on the results of the SHAPE study in women with a BMI≥25, the DIANA trial^46^ and the studies of McTiernan^13^ and Friedenreich ^15^:

- Mean log-transformed serum estradiol levels: 2.28 pg/ml
- Log-transformed SD: 0.44 pg/ml
- Change in diet group: -12%; pg/ml
- Change in exercise-weight loss group: -20%: pg/ml
- Power: minimally 80%
- Level of significance: 0.05 for comparison 1 and 0.025 for comparisons 2 and 3 (taking into account multiple testing).
- Drop-out: 5% in all groups
- Non-compliance/contamination: 15%

First, we calculated the sample size for the first comparison, i.e. diet-induced weight loss (n=85) versus exercise-induced weight loss (n=85), since the difference in estradiol levels between these groups is expected to be the smallest (8%). Furthermore, we think a difference of 8% will be of clinical relevance. Based on the estimated sample sizes, we calculated the number of subjects needed in the control group (n=36). The sample size of the control group can be much smaller, since the expected difference with the interventions groups is large (12% and 20%, respectively).

The sample size calculations resulted in the following estimated numbers per group, taking into account drop out and non-compliance:

- - Control group: 45
  - Diet group: 104
  - Combined exercise plus diet group: 104

The accompanying power for the other hormones to demonstrate an 8% difference between the diet and exercise group for is tabulated below:

|  | Diet vs. exercise |
| --- | --- |
| Estrone | 83% |
| Testosterone | 100% |
| SHBG | 96% |

*Abdominal fat measured by MRI*

Based on the study of Ross et al.^80^ (in their obese study population: mean total visceral fat was 2.3 kg SD 0.8 kg), we calculated that 81 subjects are needed to demonstrate a difference of 0.2 kg visceral fat (Alpha=0.05; Beta=0.20.) between the diet and exercise group.

# METHODS

## Study parameters/endpoints

### Main study parameter/endpoint

The primary outcome measures are serum sex-hormone levels; estradiol (total, free), estrone, testosterone, sex hormone binding globulin.

### Secondary study parameters/endpoints

The secondary measurements include BMI, weight, waist- and hip-circumference, total body fat, abdominal fat (subcutaneous and visceral), fitness (maximal exercise capacity test by the steep ramp protocol) and blood pressure.

### Other study parameters

The main confounders in this study are undesirable changes in total calorie intake and energy expenditure. From literature, we know that it is hard to regulate these factors strictly. Compensation, either unintentional or intentional, occurs quickly.

To regulate these factors to a maximum, we will monitor in several ways and give clear instructions. Monitoring of dietary intake will be performed by questionnaires and online food diaries. Monitoring of exercise behaviour will be done by questionnaires, moving sensors and heart rate monitors. Furthermore, regular measures of body weight will be performed

## Randomisation, blinding and treatment allocation

Randomisation takes place after the run-in phase. It will be performed by the department of data-management of the Julius Center (UMC Utrecht). We will use block randomisation, with a block size of 5, stratified by centrum and municipality.

## Study procedures/data collection

**Physical examination**

*Anthropometry*

- - Body weight and height

Body weight and height (to the nearest 0.5 kg and 0.5 cm respectively) will be measured while the subjects wearing light clothes and without shoes using an analogue balance (SECA) and wall-mounted tape measure.

- - Body fat distribution, i.e waist and hip circumference

Waist circumference (to the nearest 0.1 cm) is measured standing at the midway between lower ribs and iliac crest. Hip circumference (to the nearest 0.1 cm) is measured standing over the buttocks. All measurements were taken in duplicate and averaged.

- - Total body fat and body fat percentage

Total body fat and body fat percentage will be determined by using a total body DEXA scan (Lunar, Prodigy ™). A total body scan analyses body composition according to a three-compartment model: fat mass, lean tissue, and bone mineral content. The standard soft tissue analysis was performed using software supplied by the manufacturer. Total body fat will be estimated for each subject in kilograms. The DEXA scan will only be used for measurement of body composition (total body fat and muscle mass). The scan will not be diagnostically screened for pathology.

- - Intra-abdominal fat

Intra-abdominal fat will de measured by an abdominal MRI scan (Philips MR Tesla 1.5 system), using the three-point IDEAL method (described by Dixon)^81^. Visceral adipose tissue (VAT) and subcutaneous adipose tissue (SAT) will be measured by segmentation with the help of semi-automatic software. **The MRI-scan will only be used for VAT and SAT measurements for scientific research purposes and not be screened for pathology by radiologists.**

*Blood pressure*

- - Blood pressure will be measured with an automatically tonometer after participants have been sitting quietly for at least five minutes.

#### *Fitness*

- - Fitness will be defined by peak heart rate, VO2 max and maximal workload. VO_2_ max will be estimated by a maximal cycle test with the help of a (standard) ramp protocol as follows:
    - 2 minutes of rest while sitting on the ergometer for measuring resting heart rate and ECG.
    - 3 minutes of cycling without workload (warming-up)
    - From minute 6 till the peak heart rate has achieved the workload will be increased every minute by either 12.5 Watt/min, 15 Watt/min, 17.5 Watt/min or 20 Watt/min, depending on the predicted maximum Watt per subject.

Predicted maximum Watt (standard formula) =

((20.4 (cm) – 8.74*(age) – 288 – 1909) x 0.1635. Divided by 10 gives a number which will be rounded off to the nearest Watt/min.

The maximal exercise capacity test will be performed in a clinical setting. During the whole test ECG will be performed. There is safety and reanimation equipment and an alarm button available within hand reach of the supervisors. There are always two supervisors performing one test, both are trained on supervising maximal capacity tests. At least one is trained in reanimation, ECG reading (especially focusing on ischemic signs) and recognizing of stop criteria.

- *Physical activity*
  - Physical activity is measured by the ActiGraph, an activity monitor. The ActiGraph is non-invasive. It will be worn twice by all participants in the run-in and maintenance phase during 7 consecutive days.

**Blood samples**

- - Blood samples will be drawn in order to determine serum concentrations of estradiol (total, free), estrone, testosterone and sex hormone binding globulin (SHBG). Blood samples will be centrifuged and stored at -80°C within 4 hours. All samples from an individual subject will be analysed in the same batch since the batch-to-batch variation can be higher than any woman's likely change in hormones over the year^82^.Serum estrogens, testosterone and SHBG will be determined by use of commercially available double-antibody radioimmunoassay kits (Roche Cobas: SHBG-03052001, testosterone-05200067190) and LDN: estradiol US ELISA E-1900, estron FR E-2300). The laboratory ”Stichting Huisartsenlaboratorium Oost” in Velp ([www.labsho.nl](http://www.labsho.nl/)) will perform all assays.

**Questionnaires/interviews**

#### *General*

- - A self-constructed questionnaire (general questionnaire) will be used to determine socio-demographic variables, smoking history, medical history and reproductive factors

#### *Physical activity*

- - Current level of physical activity is measured via the Short-Questionnaire to Assess Health-enhancing physical activity (SQUASH).^83^ This questionnaire will be filled in once by all participants.
  - Life-time physical activity will be measured via a questionnaire designed by Friedenreich et al. This questionnaire will be filled in once by all participants.^84^
  - To be able to measure changes in physical activity during the study the PASE-questionnaire is used. (Physical Activity Scale for the Elderly).^85^ This questionnaire will be filled in twice by all participants.

#### *Diet*

- - Daily food intake will be assessed by the dietician in a one-hour individual session. The focus will be on (total) calorie intake, macronutrients, fibre and alcohol intake
  - An online food-diary on the Dutch website [www.dieeninzicht.nl](http://www.dieeninzicht.nl/) will be used to monitor dietary intake over 3 consecutive days during the study. This diary includes information on macronutrients, dietary fibre and total amount of calories. This diary will be filled in three times during the study period by all participants.

#### *Medication use*

- - Medication use will be asked during the screening- and measurement visits and registered by use of an electronic database.

**Incidental findings**

Measurements will be reviewed on study outcome parameters only and can not be seen as a medical screening. However, incidental findings can arise in the different measurements, as high blood glucose level or deviations on the exercise ECG, these will always be reported to the subjects.

MRI-scan: MRI scans are not reviewed by a radiologist. N**ew MRI-scans will be monthly screened by the SHAPE-2 researchers. And at the end of the study during the VAT/SAT measurement procedure. In case of clearly visible incidental findings the scans will be reviewed by a radiologist and the subject will be informed.**

## Withdrawal of individual subjects

Subjects can leave the study at any time for any reason if they wish to do so without any consequences. The investigator can decide to withdraw a subject from the study for urgent medical reasons or in case the subject is not compliant to the inclusion criteria or study intervention (confirmed or with high suspicion).

# SAFETY REPORTING

## Section 10 WMO event

In accordance to section 10, subsection 1, of the WMO, the investigator will inform the subjects and the reviewing accredited METC if anything occurs, on the basis of which it appears that the disadvantages of participation may be significantly greater than was foreseen in the research proposal. The study will be suspended pending further review by the accredited METC, except insofar as suspension would jeopardise the subjects’ health. The investigator will take care that all subjects are kept informed.

## Adverse and serious adverse events

Adverse events are defined as any undesirable experience occurring to a subject during the study, potentially related to the intervention programme or study measures. All adverse events reported spontaneously by the subject or observed by the investiga­tor or his staff will be recorded in an electronic database.

A serious adverse event is any untoward medical occurrence or effect that at any dose results in death;

- is life threatening (at the time of the event);
- requires hospitalisation or prolongation of existing inpatients’ hospitalisation;
- results in persistent or significant disability or incapacity;
- is a new event of the trial likely to affect the safety of the subjects, such as an unexpected outcome of an adverse reaction, lack of efficacy of an IMP used for the treatment of a life threatening disease, major safety finding from a newly completed animal study, etc.

SAEs will be reported at the end of the study to the accredited METC that approved the protocol. However, SAEs that result in death or are life threatening will be reported expedited through the webportal Toetsingonline. The expedited reporting will occur not later than 7 days after the responsible investigator has first knowledge of the adverse reaction. This is for a preliminary report with another 8 days for completion of the report.

## Follow-up of adverse events

In case of non-attendance to sports classes, dietary (group) sessions or planned visits to the hospital the reasons for absence will be checked (by dietician/physiotherapist) and be reported to the researchers.

All participants are asked to report unplanned hospitalisation during the study period; this will also be checked at the end of the study.

# STATISTICAL ANALYSIS

## Descriptive statistics

Baseline demographics and characteristics will be reported by treatment group descriptively. Means and SDs will be presented for continuous normally distributed variables and medians and ranges for non-normally distributed variables. Categorical variables will be presented by frequencies per group (absolute numbers and percentages).

Baseline and end of study values of sex hormone levels, total body fat and intra-abdominal body fat will be tabulated by treatment group. If sex hormone levels are non-normally distributed, these will be log transformed and geometric means will be presented.

## Analysis

Our primary and secondary endpoints will be changes in sex steroid hormones, body composition and fitness during the study. If sex hormones are non-normally distributed, log transformations will be used for analyses. We will analyse the effects by linear regression analysis. We will compare the effects of the diet group versus the exercise group, the diet versus control, and exercise versus control. The main analysis will be performed according to the intention-to-treat principle, where outcomes for patients are analyzed by assigned treatment, regardless of the level of adherence. As a secondary analysis, adherence will be examined as a potential modifier of the intervention effects.

To explore whether changes in body fat (total, abdominal) mediates intervention effects on sex hormone levels, we examined the change in the measure of association pre- versus post adjustment for changes in body fat (in the linear regression model). If the adjustment attenuates the measure of association, then we consider this to be suggestive of mediation. Furthermore, the main effect of total body fat/ abdominal fat on changes in sex hormone levels will also be evaluated in the linear regression models, adjusting for the intervention assignment. This method is based on a paper of Kraemer et al^86^ which explains that a mediator must show a change during treatment, must correlate with the treatment assignment, and must have a main or interactive effect on the outcome.

# ETHICAL CONSIDERATIONS

## Regulation statement

This study will be conducted according to the principles of the Declaration of Helsinki (version 59, October 2008) and in accordance with the Medical Research Involving Human Subjects Act (WMO).

## Recruitment and consent

Women will be recruited through a random selection out of the female population aging 50-69 years in the surroundings of Utrecht and Enschede. For their contact details, we will use the community register (GBA) and the Julius-UMCU register with potential candidates for scientific research (POKA).

First, potential candidates receive an invitation letter by mail explaining the goal of the study and a short eligibility questionnaire. In the invitation letter, we also provide a link to an online version of the information brochure for participants. All women are asked to return the reply card on which they can indicate their interest.

Second, interested women will be contacted by phone to explain further details and to screen in- and exclusion criteria. For eligible women who are still interested, an appointment will be made for a screening and informed consent visit. We will send these women a paper copy of the information brochure including informed consent form and a questionnaire concerning their physical activity pattern.

At the screening visit, informed consent will be signed first. Thereafter, women will be screened for eligibility by history taking, checking the completed questionnaire, physical examination and a glucose check (to rule out an increased blood glucose level or diabetes mellitus). For eligible women, an intake visit by the dietician will be planned.

The time interval between receiving the paper copy of the information brochure and the planned screening visit will be at least 1 week so that potential participants have their respite.

**Mailing**

Information brochure including informed consent

Appointment confirmation + itinerary

**Telephonic contact**

- Further assess eligibility of interested candidates:

🡪 Check again: BMI, smoking, postmenopausal status,

history of cancer/diabetes/endocrinal diseases.

🡪 Plus: physical activity, medication/supplements use,

hormone use, availability for sports/dietician contacts.

Make appointment for screening visit

**Identification of potential eligible participants from:**

- Governmental citizen register (GBA)

- Possible candidates register (POKA)

**Mailing**

**-** Brief invitation letter including:

- Inclusion criteria

- A link to an online information brochure

- Reply-card

**Screening visit**

**(After a minimal time interval of 1 week)**

Handing in informed consent

BMI, Glucose check, medication and hormone use.

Plan visit dietician for dietary intake

*n= 271**

***Figure 2. Flow-chart recruitment of participants***

Participants are equally recruited in the UMCU and MST region

## Compensation for injury

The sponsor/investigator has a liability insurance which is in accordance with article 7, subsection 6 of the WMO and the Measure regarding Compulsory Insurance for Clinical Research in Humans of 23th June 2003). This insurance provides cover for damage to research subjects through injury or death caused by the study.

1. € 450.000,-- (i.e. four hundred and fifty thousand Euro) for death or injury for each subject who participates in the Research;
2. € 3.500.000,-- (i.e. three million five hundred thousand Euro) for death or injury for all subjects who participate in the Research;
3. € 5.000.000,-- (i.e. five million Euro) for the total damage incurred by the organisation for all damage disclosed by scientific research for the Sponsor as ‘verrichter’ in the meaning of said Act in each year of insurance coverage.

The insurance applies to the damage that becomes apparent during the study or within 4 years after the end of the study.

## Incentives

Travel expenses for hospital visits for eligibility screening, physical examination, MRI- and DEXA-scanning and blood collection will be compensated.

# ADMINISTRATIVE ASPECTS AND PUBLICATION

## Handling and storage of data and documents

All study data will be handled confidentially. Personal data will be coded and the key of this code will be safeguarded by the principal investigator. This code will not be based on the participant’s initials and birth-date. The researchers will handle all data complying with the Dutch Personal Data Protection Act (in Dutch: De Wet Bescherming Persoonsgegevens, Wbp).

All data and human material acquired in the study will be stored for a maximum of 20 years after the end of the study. Personal details (name and address details) will be destroyed after this period.

## Monitoring

The SHAPE-2 study will be checked on safety for subjects and validity of data by an independent monitor as described in the Monitoring Plan designed for the SHAPE-2 study. The Monitoring Plan meets the requirements of the guidelines for study monitoring of the Dutch Federation of University Medical (NFU) and the UMCU/Julius Center. The Monitoring Plan has been approved by the Bureau Quality Assurance (BKO) of the UMCU.

## Amendments

Amendments are changes made to the research after a favourable opinion by the accredited METC has been given. All amendments will be notified to the METC that gave a favourable opinion.

Non-substantial amendments will not be notified to the accredited METC and the competent authority, but will be recorded and filed by the sponsor.

## Annual progress report

The sponsor/investigator will submit a summary of the progress of the trial to the accredited METC once a year. Information will be provided on the date of inclusion of the first subject, numbers of subjects included and numbers of subjects that have completed the trial, serious adverse events/ serious adverse reactions, other problems, and amendments.

## End of study report

The investigator will notify the accredited METC of the end of the study within a period of 8 weeks. The end of the study is defined as the last patient’s last visit.

In case the study is ended prematurely, the investigator will notify the accredited METC, including the reasons for the premature termination.

Within one year after the end of the study, the investigator/sponsor will submit a final study report with the results of the study, including any publications/abstracts of the study, to the accredited METC.

## Public disclosure and publication policy

The SHAPE-2 trial will be registered in a public trial registry. We agree on the CCMO’s position on the disclosure/publication of the research results obtained from studies involving human subjects. The research data will be disclosed unreservedly by the investigators.

# REFERENCES

1. Weight control and physical activity. Lyon: IARC Press; 2002.

2. Monninkhof EM, Elias SG, Vlems FA et al. Physical activity and breast cancer: a systematic review. Epidemiology 2007; 18(1):137-157.

3. Campbell KL, McTiernan A. Exercise and biomarkers for cancer prevention studies. J Nutr 2007; 137(1):161S-169S.

4. World Cancer Research Fund, American Institute for Cancer Research. Food, Nutrition, Physical Activity, and the Prevention of Cancer: a Global Perspective. 2007. Washington DC, AICR.
Ref Type: Report

5. Neilson HK, Friedenreich CM, Brockton NT, Millikan RC. Physical activity and postmenopausal breast cancer: proposed biologic mechanisms and areas for future research. Cancer Epidemiol Biomarkers Prev 2009; 18(1):11-27.

6. Renehan AG, Tyson M, Egger M, Heller RF, Zwahlen M. Body-mass index and incidence of cancer: a systematic review and meta-analysis of prospective observational studies. Lancet 2008; 371(9612):569-578.

7. Key TJ, Appleby PN, Reeves GK et al. Body mass index, serum sex hormones, and breast cancer risk in postmenopausal women. J Natl Cancer Inst 2003; 95(16):1218-1226.

8. Eliassen AH, Colditz GA, Rosner B, Willett WC, Hankinson SE. Adult weight change and risk of postmenopausal breast cancer. JAMA 2006; 296(2):193-201.

9. Harvie M, Howell A, Vierkant RA et al. Association of gain and loss of weight before and after menopause with risk of postmenopausal breast cancer in the Iowa women's health study. Cancer Epidemiol Biomarkers Prev 2005; 14(3):656-661.

10. Trentham-Dietz A, Newcomb PA, Egan KM et al. Weight change and risk of postmenopausal breast cancer (United States). Cancer Causes Control 2000; 11(6):533-542.

11. Eng SM, Gammon MD, Terry MB et al. Body size changes in relation to postmenopausal breast cancer among women on Long Island, New York. Am J Epidemiol 2005; 162(3):229-237.

12. McTiernan A, Tworoger SS, Rajan KB et al. Effect of exercise on serum androgens in postmenopausal women: a 12-month randomized clinical trial. Cancer Epidemiol Biomarkers Prev 2004; 13(7):1099-1105.

13. McTiernan A, Tworoger SS, Ulrich CM et al. Effect of exercise on serum estrogens in postmenopausal women: a 12-month randomized clinical trial. Cancer Res 2004; 64(8):2923-2928.

14. Monninkhof EM, Velthuis MJ, Peeters PH, Twisk J, Schuit AJ. The effect of exercise on postmenopausal sex hormone levels and the role of body fat: a randomized controlled trial. J Clin Oncol 2009; 27.

15. Friedenreich CM, Woolcott CG, McTiernan A et al. Alberta physical activity and breast cancer prevention trial: sex hormone changes in a year-long exercise intervention among postmenopausal women. J Clin Oncol 2010; 28(9):1458-1466.

16. Velthuis MJ, Schuit AJ, Peeters PH, Monninkhof EM. Exercise program affects body composition but not weight in postmenopausal women. Menopause 2009; 16(4):777-784.

17. Foster-Schubert KE, Alfano CM, Duggan CR et al. Effect of Diet and Exercise, Alone or Combined, on Weight and Body Composition in Overweight-to-Obese Postmenopausal Women. Obesity (Silver Spring) 2011.

18. King NA, Caudwell P, Hopkins M et al. Metabolic and behavioral compensatory responses to exercise interventions: barriers to weight loss. Obesity (Silver Spring) 2007; 15(6):1373-1383.

19. Blundell JE, Stubbs RJ, Hughes DA, Whybrow S, King NA. Cross talk between physical activity and appetite control: does physical activity stimulate appetite? Proc Nutr Soc 2003; 62(3):651-661.

20. Hankinson SE, Eliassen AH. Endogenous estrogen, testosterone and progesterone levels in relation to breast cancer risk. J Steroid Biochem Mol Biol 2007; 106(1-5):24-30.

21. Kaaks R, Rinaldi S, Key TJ et al. Postmenopausal serum androgens, oestrogens and breast cancer risk: the European prospective investigation into cancer and nutrition. Endocr Relat Cancer 2005; 12(4):1071-1082.

22. Key T, Appleby P, Barnes I, Reeves G. Endogenous sex hormones and breast cancer in postmenopausal women: reanalysis of nine prospective studies. J Natl Cancer Inst 2002; 94(8):606-616.

23. Tamimi RM, Byrne C, Colditz GA, Hankinson SE. Endogenous hormone levels, mammographic density, and subsequent risk of breast cancer in postmenopausal women. J Natl Cancer Inst 2007; 99(15):1178-1187.

24. Nelson ME, Meredith CN, Dawson-Hughes B, Evans WJ. Hormone and bone mineral status in endurance-trained and sedentary postmenopausal women. J Clin Endocrinol Metab 1988; 66(5):927-933.

25. McTiernan A, Wu L, Chen C et al. Relation of BMI and physical activity to sex hormones in postmenopausal women. Obesity (Silver Spring) 2006; 14(9):1662-1677.

26. Cauley JA, Gutai JP, Kuller LH, LeDonne D, Powell JG. The epidemiology of serum sex hormones in postmenopausal women. Am J Epidemiol 1989; 129(6):1120-1131.

27. Chan MF, Dowsett M, Folkerd E et al. Usual physical activity and endogenous sex hormones in postmenopausal women: the European prospective investigation into cancer-norfolk population study. Cancer Epidemiol Biomarkers Prev 2007; 16(5):900-905.

28. Schmitz KH, Lin H, Sammel MD et al. Association of physical activity with reproductive hormones: the Penn Ovarian Aging Study. Cancer Epidemiol Biomarkers Prev 2007; 16(10):2042-2047.

29. Madigan MP, Troisi R, Potischman N, Dorgan JF, Brinton LA, Hoover RN. Serum hormone levels in relation to reproductive and lifestyle factors in postmenopausal women (United States). Cancer Causes Control 1998; 9(2):199-207.

30. Van Gils CH, Peeters PH, Schoenmakers MC et al. Physical activity and endogenous sex hormone levels in postmenopausal women: a cross-sectional study in the Prospect-EPIC Cohort. Cancer Epidemiol Biomarkers Prev 2009; 18(2):377-383.

31. Bjornerem A, Straume B, Midtby M et al. Endogenous sex hormones in relation to age, sex, lifestyle factors, and chronic diseases in a general population: the Tromso Study. J Clin Endocrinol Metab 2004; 89(12):6039-6047.

32. Verkasalo PK, Thomas HV, Appleby PN, Davey GK, Key TJ. Circulating levels of sex hormones and their relation to risk factors for breast cancer: a cross-sectional study in 1092 pre- and postmenopausal women (United Kingdom). Cancer Causes Control 2001; 12(1):47-59.

33. Siiteri PK. Adipose tissue as a source of hormones. Am J Clin Nutr 1987; 45(1 Suppl):277-282.

34. Judd HL, Shamonki IM, Frumar AM, Lagasse LD. Origin of serum estradiol in postmenopausal women. Obstet Gynecol 1982; 59(6):680-686.

35. Key TJ, Allen NE, Verkasalo PK, Banks E. Energy balance and cancer: the role of sex hormones. Proc Nutr Soc 2001; 60(1):81-89.

36. Lukanova A, Lundin E, Zeleniuch-Jacquotte A et al. Body mass index, circulating levels of sex-steroid hormones, IGF-I and IGF-binding protein-3: a cross-sectional study in healthy women. Eur J Endocrinol 2004; 150(2):161-171.

37. Hankinson SE, Willett WC, Manson JE et al. Alcohol, height, and adiposity in relation to estrogen and prolactin levels in postmenopausal women. J Natl Cancer Inst 1995; 87(17):1297-1302.

38. Wu F, Ames R, Evans MC, France JT, Reid IR. Determinants of sex hormone-binding globulin in normal postmenopausal women. Clin Endocrinol (Oxf) 2001; 54(1):81-87.

39. Austin H, Austin JM, Jr., Partridge EE, Hatch KD, Shingleton HM. Endometrial cancer, obesity, and body fat distribution. Cancer Res 1991; 51(2):568-572.

40. Bezemer ID, Rinaldi S, Dossus L et al. C-peptide, IGF-I, sex-steroid hormones and adiposity: a cross-sectional study in healthy women within the European Prospective Investigation into Cancer and Nutrition (EPIC). Cancer Causes Control 2005; 16(5):561-572.

41. Katsouyanni K, Boyle P, Trichopoulos D. Diet and urine estrogens among postmenopausal women. Oncology 1991; 48(6):490-494.

42. Kaye SA, Folsom AR, Soler JT, Prineas RJ, Potter JD. Associations of body mass and fat distribution with sex hormone concentrations in postmenopausal women. Int J Epidemiol 1991; 20(1):151-156.

43. Newcomb PA, Klein R, Klein BE et al. Association of dietary and life-style factors with sex hormones in postmenopausal women. Epidemiology 1995; 6(3):318-321.

44. de Waard F, Poortman J, Pedro-Alvarez FM, Baanders-van Halewijn EA. Weight reduction and oestrogen excretion in obese post-menopausal women. Maturitas 1982; 4(2):155-162.

45. Wu AH, Pike MC, Stram DO. Meta-analysis: dietary fat intake, serum estrogen levels, and the risk of breast cancer. J Natl Cancer Inst 1999; 91(6):529-534.

46. Berrino F, Bellati C, Secreto G et al. Reducing bioavailable sex hormones through a comprehensive change in diet: the diet and androgens (DIANA) randomized trial. Cancer Epidemiol Biomarkers Prev 2001; 10(1):25-33.

47. Borm GF, Hoogendoorn EH, den HM, Zielhuis GA. Sequential balancing: a simple method for treatment allocation in clinical trials. Contemp Clin Trials 2005; 26(6):637-645.

48. Roza AM, Shizgal HM. The Harris Benedict equation reevaluated: resting energy requirements and the body cell mass. Am J Clin Nutr 1984; 40(1):168-182.

49. Kwaliteitsinstituut voor de Gezondheidszorg CBO, Nederlands Huisartsen Genootschap. Multidisciplinaire richtlijn: Cardiovasculair risicomanagement 2006. 2006. Alphen aan den Rijn, Van Zuiden Communications B.V.
Ref Type: Report

50. The Health Counsil of The Netherlands. Richtlijnen goede voeding 2006 -achtergronddocument. A06/08. 2006. The Hague.
Ref Type: Report

51. Riedt CS, Cifuentes M, Stahl T, Chowdhury HA, Schlussel Y, Shapses SA. Overweight postmenopausal women lose bone with moderate weight reduction and 1 g/day calcium intake. J Bone Miner Res 2005; 20(3):455-463.

52. Shapses SA, Riedt CS. Bone, body weight, and weight reduction: what are the concerns? J Nutr 2006; 136(6):1453-1456.

53. Hardeman W, Griffin S, Johnston M, Kinmonth AL, Wareham NJ. Interventions to prevent weight gain: a systematic review of psychological models and behaviour change methods. Int J Obes Relat Metab Disord 2000; 24(2):131-143.

54. Britt E, Hudson SM, Blampied NM. Motivational interviewing in health settings: a review. Patient Educ Couns 2004; 53(2):147-155.

55. Prochaska JO, Velicer WF. The transtheoretical model of health behavior change. Am J Health Promot 1997; 12(1):38-48.

56. Avenell A, Sattar N, Lean M. ABC of obesity. Management: Part I--behaviour change, diet, and activity. BMJ 2006; 333(7571):740-743.

57. Fabricatore AN. Behavior therapy and cognitive-behavioral therapy of obesity: is there a difference? J Am Diet Assoc 2007; 107(1):92-99.

58. Foster G. The behavioral approach to treating obesity. Am Heart J 2006; 151(3):625-627.

59. Lang A, Froelicher ES. Management of overweight and obesity in adults: behavioral intervention for long-term weight loss and maintenance. Eur J Cardiovasc Nurs 2006; 5(2):102-114.

60. Jones LR, Wilson CI, Wadden TA. Lifestyle modification in the treatment of obesity: an educational challenge and opportunity. Clin Pharmacol Ther 2007; 81(5):776-779.

61. Renjilian DA, Perri MG, Nezu AM, McKelvey WF, Shermer RL, Anton SD. Individual versus group therapy for obesity: effects of matching participants to their treatment preferences. J Consult Clin Psychol 2001; 69(4):717-721.

62. Kwaliteitsinstituut voor de Gezondheidszorg CBO. Richtlijn: Diagnostiek en behandeling van obesitas
bij volwassenen en kinderen. 2008. Alphen aan den Rijn, Van Zuiden Communications B.V.
Ref Type: Report

63. The Diabetes Prevention Program (DPP): description of lifestyle intervention. Diabetes Care 2002; 25(12):2165-2171.

64. Dansinger ML, Tatsioni A, Wong JB, Chung M, Balk EM. Meta-analysis: the effect of dietary counseling for weight loss. Ann Intern Med 2007; 147(1):41-50.

65. Wadden TA, Butryn ML. Behavioral treatment of obesity. Endocrinol Metab Clin North Am 2003; 32(4):981-1003, x.

66. Sacks FM, Bray GA, Carey VJ et al. Comparison of weight-loss diets with different compositions of fat, protein, and carbohydrates. N Engl J Med 2009; 360(9):859-873.

67. Knechtle B, Muller G, Willmann F, Kotteck K, Eser P, Knecht H. Fat oxidation in men and women endurance athletes in running and cycling. Int J Sports Med 2004; 25(1):38-44.

68. Asikainen TM, Kukkonen-Harjula K, Miilunpalo S. Exercise for health for early postmenopausal women: a systematic review of randomised controlled trials. Sports Med 2004; 34(11):753-778.

69. Stiegler P, Cunliffe A. The role of diet and exercise for the maintenance of fat-free mass and resting metabolic rate during weight loss. Sports Med 2006; 36(3):239-262.

70. Kohrt WM, Spina RJ, Holloszy JO, Ehsani AA. Prescribing exercise intensity for older women. J Am Geriatr Soc 1998; 46(2):129-133.

71. Ainsworth BE, Haskell WL, Leon AS et al. Compendium of physical activities: classification of energy costs of human physical activities. Med Sci Sports Exerc 1993; 25(1):71-80.

72. Ainsworth BE, Haskell WL, Whitt MC et al. Compendium of physical activities: an update of activity codes and MET intensities. Med Sci Sports Exerc 2000; 32(9 Suppl):S498-S504.

73. Byrne NM, Hills AP, Hunter GR, Weinsier RL, Schutz Y. Metabolic equivalent: one size does not fit all. J Appl Physiol 2005; 99(3):1112-1119.

74. Kozey S, Lyden K, Staudenmayer J, Freedson P. Errors in MET estimates of physical activities using 3.5 ml x kg(-1) x min(-1) as the baseline oxygen consumption. J Phys Act Health 2010; 7(4):508-516.

75. Monninkhof EM, Velthuis MJ, Peeters PH, Twisk JW, Schuit AJ. Effect of exercise on postmenopausal sex hormone levels and role of body fat: a randomized controlled trial. J Clin Oncol 2009; 27(27):4492-4499.

76. AJ Schuit. Regular Physical activity in old age. Effect on coronary heart disease risk factors and well-being. 1997.

77. Figard-Fabre H, Fabre N, Leonardi A, Schena F. Physiological and perceptual responses to Nordic walking in obese middle-aged women in comparison with the normal walk. Eur J Appl Physiol 2010; 108(6):1141-1151.

78. Miller NE, Strath SJ, Swartz AM, Cashin SE. Estimating absolute and relative physical activity intensity across age via accelerometry in adults. J Aging Phys Act 2010; 18(2):158-170.

79. CBS Statistics Netherlands. [Lifestyle and preventive behaviour, body length and weight]. 5-9-2011.
Ref Type: Data File

80. Ross R, Janssen I, Dawson J et al. Exercise-induced reduction in obesity and insulin resistance in women: a randomized controlled trial. Obes Res 2004; 12(5):789-798.

81. Dixon WT. Simple proton spectroscopic imaging. Radiology 1984; 153(1):189-194.

82. Tworoger SS, Yasui Y, Chang L, Stanczyk FZ, McTiernan A. Specimen allocation in longitudinal biomarker studies: controlling subject-specific effects by design. Cancer Epidemiol Biomarkers Prev 2004; 13(7):1257-1260.

83. Wendel-Vos GC, Schuit AJ, Saris WH, Kromhout D. Reproducibility and relative validity of the short questionnaire to assess health-enhancing physical activity. J Clin Epidemiol 2003; 56(12):1163-1169.

84. Friedenreich CM, Courneya KS, Bryant HE. The lifetime total physical activity questionnaire: development and reliability. Med Sci Sports Exerc 1998; 30(2):266-274.

85. Schuit AJ, Schouten EG, Westerterp KR, Saris WH. Validity of the Physical Activity Scale for the Elderly (PASE): according to energy expenditure assessed by the doubly labeled water method. J Clin Epidemiol 1997; 50(5):541-546.

86. Kraemer HC, Wilson GT, Fairburn CG, Agras WS. Mediators and moderators of treatment effects in randomized clinical trials. Arch Gen Psychiatry 2002; 59(10):877-883.
